# Immunoproteasome function maintains oncogenic gene expression in KMT2A-complex driven leukemia

**DOI:** 10.1186/s12943-023-01907-7

**Published:** 2023-12-04

**Authors:** Nuria Tubío-Santamaría, Ashok Kumar Jayavelu, Tina M. Schnoeder, Theresa Eifert, Chen-Jen Hsu, Florian Perner, Qirui Zhang, Daniela V. Wenge, Fynn M. Hansen, Joanna M. Kirkpatrick, Nidhi Jyotsana, Steven W. Lane, Björn von Eyss, Aniruddha J. Deshpande, Michael W. M. Kühn, Juerg Schwaller, Clemens Cammann, Ulrike Seifert, Frédéric Ebstein, Elke Krüger, Andreas Hochhaus, Michael Heuser, Alessandro Ori, Matthias Mann, Scott A. Armstrong, Florian H. Heidel

**Affiliations:** 1 Innere Medizin C, Universitätsmedizin Greifswald, 17475 Greifswald, Germany; 2 Leibniz Institute On Aging, Fritz-Lipmann Institute, 07745 Jena, Germany; 3 Max-Planck-Institute of Biochemistry, Munich, Germany; 4 Proteomics and Cancer Cell Signaling Group, DKFZ, Heidelberg, Germany; 5 Department of Pediatric Oncology, Dana Farber Cancer Institute, Harvard University, Boston, MA 02215 USA; 6 Independent consultant, London, UK; 7 Department of Cell and Developmental Biology, Vanderbilt University, Nashville, TN USA; 8 Queensland Institute for Medical Research (QIMR), Brisbane, Australia; 9 Sanford Burnham Research Institute, San Diego, USA; 10 Medizinische Klinik 3, Hämatologie, Onkologie und Pneumologie, Universitätsmedizin Mainz, Mainz, Germany; 11 Department of Biomedicine, University Children’s Hospital of Basel, Basel, Switzerland; 12 Friedrich Loeffler-Institut für Medizinische Mikrobiologie - Virologie, Universitätsmedizin Greifswald, 17475 Greifswald, Germany; 13 Department of Biochemistry, Universitätsmedizin Greifswald, 17475 Greifswald, Germany; 14 Innere Medizin 2, Universitätsklinikum Jena, Jena, Germany; 15 Hematology, Hemostasis, Oncology and Stem Cell Transplantation, Hannover Medical School (MHH), Hannover, Germany

## Abstract

Pharmacologic targeting of chromatin-associated protein complexes has shown significant responses in *KMT2A*-rearranged (*KMT2A-r*) acute myeloid leukemia (AML) but resistance frequently develops to single agents. This points to a need for therapeutic combinations that target multiple mechanisms. To enhance our understanding of functional dependencies in *KMT2A-r* AML, we have used a proteomic approach to identify the catalytic immunoproteasome subunit PSMB8 as a specific vulnerability. Genetic and pharmacologic inactivation of *PSMB8* results in impaired proliferation of murine and human leukemic cells while normal hematopoietic cells remain unaffected. Disruption of immunoproteasome function drives an increase in transcription factor BASP1 which in turn represses KMT2A-fusion protein target genes. Pharmacologic targeting of PSMB8 improves efficacy of Menin-inhibitors, synergistically reduces leukemia in human xenografts and shows preserved activity against Menin-inhibitor resistance mutations. This identifies and validates a cell-intrinsic mechanism whereby selective disruption of proteostasis results in altered transcription factor abundance and repression of oncogene-specific transcriptional networks. These data demonstrate that the immunoproteasome is a relevant therapeutic target in AML and that targeting the immunoproteasome in combination with Menin-inhibition could be a novel approach for treatment of *KMT2A-r* AML.

**Supplementary Information:**

The online version contains supplementary material available at 10.1186/s12943-023-01907-7.

## Statement of significance

Resistance to targeted epigenetic therapies evolves as a clinical challenge. Identification and validation of immunoproteasome function for the first time as a cell intrinsic target across KMT2A-complex dependent leukemias creates a unique functional KMT2A-r and NPM1c AML specific dependency, limiting LSC self-renewal. This facilitates therapeutic targeting with preserved efficacy against Menin-inhibitor resistant clones.

## Introduction

Leukemias harboring translocations involving chromosome 11q23 are characterized by rearrangements of the Mixed-Lineage-Leukemia-gene (*MLL1, KMT2A*) and result in aberrant regulation of the epigenetic landscape [1]. These rearrangements involve several potential translocation partners and confer a particularly poor prognosis [2]. KMT2A-fusion proteins generated by *KMT2A*-rearrangements are part of a large multi-protein complex that is associated with chromatin and drives leukemia through deregulation of transcriptional networks. While direct targeting of the KMT2A-fusions has not been successful, recent reports indicate that indirect targeting of KMT2A-associated protein-chromatin-complexes is feasible and effective [1]. Here, pharmacologic inactivation of KMT2A-complex proteins Dot1L and Menin (MEN1) resulted in loss of self-renewal and out-competition of leukemic cells [3–7]. Interestingly, gene expression programs driven by oncogenic KMT2A-fusions are also relevant for other subtypes of leukemia, especially *NPM1*-mutant (*NPM1c*) AML [6, 8]. Translation of these findings into early clinical trials have demonstrated impressive responses but also show development of clinical resistance [9, 10]. These results emphasize the need to prevent therapy resistance and to develop drug combinations that target dysregulated transcriptional networks to improve efficacy. As oncogenes can generate secondary dependencies, targeting of these vulnerabilities may result in synthetic lethality that is oncogene specific. Our studies uncover a selective dependency of *KMT2A*-rearranged (*KMT2A-r*) leukemias on proteostasis and specifically immunoproteasome function. This interferon-induced version of the proteasome with high efficacy for basic proteins is most abundantly expressed in hematopoietic and immune cells [11–14]. Differential expression of immunoproteasome (and proteasome) subunits has been correlated with therapeutic response and sensitivity to proteasome inhibitors in leukemia [15, 16]. Recently, morphologically defined subsets of AML (FABM5) and those with KMT2A rearrangements have been shown to upregulate immunoproteasome genes in a cell-intrinsic manner in order to resist cell stress [17]. Consistent with this finding, activity of immunoproteasome inhibitors was described in two acute lymphoblastic leukemia (ALL) cell lines harboring KMT2A::AFF1 (MLL-AF4) fusions [18].

Our studies uncover a selective dependency of all *KMT2A*-complex dependent (KMT2A-r and NPM1c) leukemias, across phenotypic subtypes, on immunoproteasome function. We describe immunoproteasome function—as executed by its catalytic subunit PSMB8—as a tractable target that is required to maintain homeostasis of transcription factor abundance. We identify Brain Abundant Membrane Attached Signal Protein 1 (BASP1) as a transcriptional repressor of KMT2A- and NPM1c-target genes that increases in abundance upon inhibition of immunoproteasome function. Combined targeting of PSMB8 and Menin leads to synergistic anti-proliferative effects, eradicates *KMT2A-r* leukemia in patient-derived xenografts and prevents development of Menin-inhibitor resistance in AML.

## Results

### Proteostasis is a unique vulnerability in KMT2A-r leukemia

**Fig. 1:**
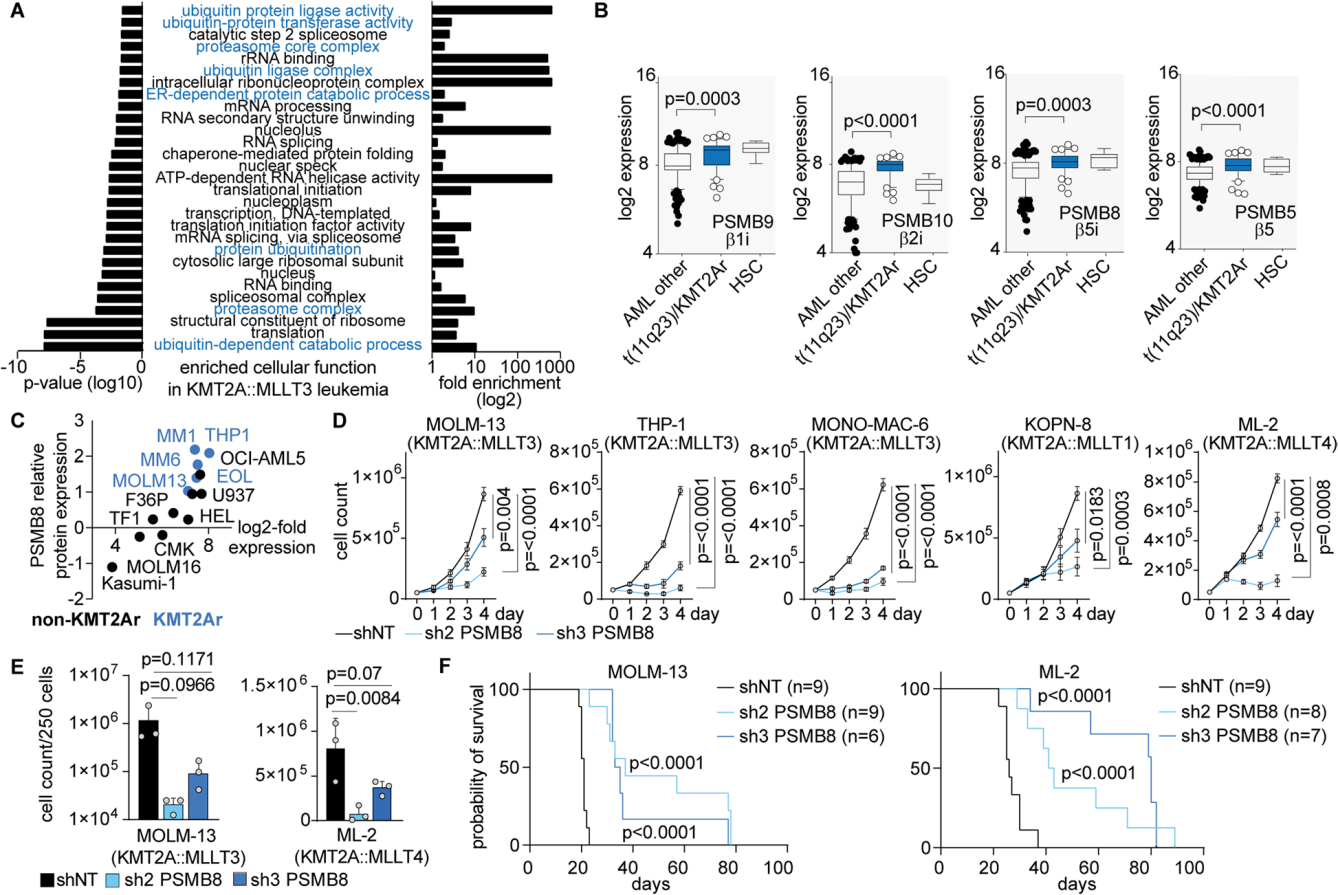
Immunoproteasome function is a vulnerability in KMT2A-r AML. **A** GO enrichment analysis on MS-based global proteome analysis on murine LSC-enriched GFP^+^ c-Kit^+^ cells of KMT2A::MLLT3-induced leukemia compared to AML1-ETO9a (*n* = 4). Displayed are the Top 30 GO-terms (“molecular function”) sorted by *p*-value. **B** Gene expression of catalytic proteasome subunits in hematopoietic stem cells (HSC), KMT2A-r leukemia (AML t(11q23)/KMT2A) or non-KMT2A-r AML (AML other) (https://servers.binf.ku.dk/bloodspot/). The box-and-whisker plots display the 90/10 percentiles at the whiskers, the 75/25 percentiles at the boxes, and the median. Mann–Whitney U test was performed. **C** Protein abundance of catalytic immunoproteasome subunit PSMB8 in KMT2A-r (blue) or non-KMT2A-r (black) AML cell lines (https://depmap.org/portal/). **D** Growth curves depicting cell counting after trypan blue exclusion of MOLM-13, THP-1, MONO-MAC-6, KOPN-8 and ML-2 cells transduced with shRNAs targeting PSMB8 or a non-targeting control (shNT). *n* = 3–5 independent experiments, in triplicate; mean with Standard Error of Mean (SEM); 2-way ANOVA. **E** Cell numbers on day 10; plating of 2.5 × 10^2^ MOLM-13 or ML-2 cells in methylcellulose. *n* = 3 independent experiments; mean with Standard Deviation (SD); paired Student t test. **F** Kaplan–Meier survival curves of NSGS recipient mice transplanted with 1 × 10^5^ MOLM-13 or ML-2 cells, expressing PSMB8-shRNA2 (*n* = 9 for MOLM-13; *n* = 8 for ML-2) and -shRNA3 (*n* = 6; *n* = 7) or non-targeting control (shNT: *n* = 9; *n* = 9); two independent cohorts; Mantel-Cox test

To assess for specific cellular functions required for fusion-oncogene driven AML and to identify oncogenic cellular functions with relevance for *KMT2A-r* leukemia, we performed global proteome profiling on either KMT2A::MLLT3 (MLL-AF9; MA9) or AML1-ETO9a (AE) AMLs generated by expression of the fusion oncogenes in murine HSPCs (Lineage^−^ Sca1^+^ c-Kit^+^, LSK). MA9 and AE induce leukemic self-renewal but have different dependency profiles as a result of different mechanisms of transformation. Cells isolated from 4 different primary recipient hosts were analyzed by in-depth quantitative proteomic analysis using high-resolution mass spectrometry (MS) [19]. The analysis determined that 868 proteins (FDR < 0.05) have differential abundance between AE- and MA9 LSC-enriched (GFP^+^ c-Kit^+^) populations. Gene set enrichment analyses (GSEA) revealed a significant enrichment of cellular functions related to proteostasis such as protein degradation and proteasome function (Fig. 1A) in the MA9 AML cells. Expression of proteasome subunits is highly heterogeneous between different cell types [12] and may also be influenced by the underlying oncogene. To investigate the expression of relevant catalytic proteasome subunits, we analyzed transcriptional regulation in published datasets. Here, catalytic subunits of the standard proteasome (PSMB5) and immunoproteasome (IP) PSMB8, PSMB9 and PSMB10 (corresponding to murine LMP7, LMP2 and LMP10) showed significantly higher expression in *KMT2A-r* AML (Fig. 1B) compared to non- *KMT2A-r* -AMLs. Likewise, *KMT2A-r* cells showed increased protein abundance of PSMB8 (Fig. 1C, Supplementary Figure S1A-B). In order to assess the functional relevance of catalytic immunoproteasome components in human AML, we performed a CRISPR-Cas9 based negative selection screen. Here, *KMT2A-r* cells showed a relevant gene-dependency specifically on PSMB8 (Supplementary Figure S1C). To validate these findings, we performed genetic suppression by RNAi with two independent shRNAs against PSMB8 (sh2- and sh3-PSMB8) compared to non-targeting control (shNT) in *KMT2A-r* cell lines. Cell counts assessed on days 1–4 revealed attenuated cell growth in *PSMB8*-suppressed cells (Fig. 1D) with variable degree of apoptosis induction contributing to this phenotype (Supplementary Figure S1D). Of note, lentiviral re-expression of codon-optimized PSMB8 blunted the response and confirmed specificity of PSMB8-dependencies (Supplementary Figure S1E). Consistently, colony forming capacity of MOLM-13 and ML-2 cells in methylcellulose was impaired following suppression of *PSMB8* (Fig. 1E, Supplementary Figure S1F). To corroborate our findings and to confirm the functional relevance of PSMB8 in vivo, we injected equal numbers of *PSMB8* depleted human AML cells into pre-conditioned (2 Gy, single dose irradiation) immuno-compromised mice (Fig. 1F, Supplementary Figure S1G). Recipients of *PSMB8* deficient cells revealed a delay in disease development and prolonged survival (median survival MOLM-13 for sh2-PSMB8: 37 days and sh3-PSMB8: 34 days; ML-2 for sh2-PSMB8: 42 days and sh3-PSMB8: 80 days) compared to NT-control (median survival MOLM-13: 21 days, *p* < 0.0001 and ML-2: 26 days; *p* < 0.0001).

**Fig. 2:**
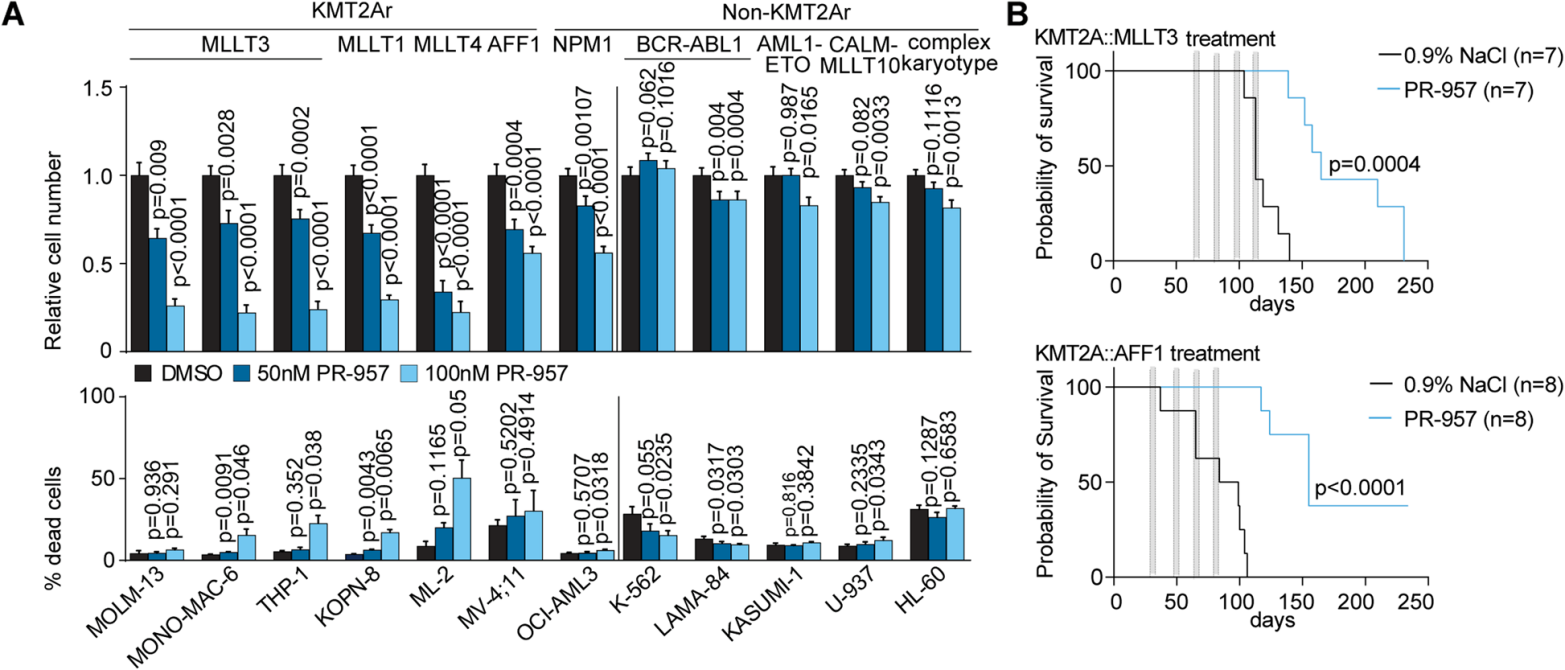
Pharmacologic targeting of PSMB8 confirms immunoproteasome requirement in KMT2A-r AML. **A** Relative cell number and percentage of dead cells (SYTOX®Blue^+^) of human leukemic cell lines as indicated after treatment with PR-957 (50 nM, 100 nM) for 96 h or DMSO as diluent control. *n* = 4 independent experiments, in triplicate; mean with SEM; paired Student t test. **B** Kaplan–Meier survival curves for two patient derived xenografts (PDX) using 1–5 × 10^4^ cells from patient samples containing an KMT2A::MLLT3 (upper) or KMT2A::AFF1 (lower) translocation injected into NSGW41 recipient mice. Recipients treated in vivo with 3-6 mg/kg PR-957 for 5 days/week or NaCl 0.9% as diluent control on 4 alternate weeks (*n* = 7 per group for KMT2A::MLLT3; *n* = 8 for KMT2A::AFF1). Two independent cohorts; Mantel-Cox test

Recently, pharmacologic targeting of specific immunoproteasome subunits has been reported [20]. The cell permeable and PSMB8 (b5i) specific compound PR-957 (ONX-0914) selectively inhibits murine and human immunoproteasome function. At nanomolar concentrations, the inhibitor does not target other standard- or immunoproteasome subunits [20, 21]. To test whether the observed dependency on PSMB8 is attributed to the loss of protein expression or rather its function, we used PR-957 on an extended panel of *KMT2A-r* versus non-*KMT2A-r* cell lines (Fig. 2A). Pharmacologic inactivation of PSMB8 resulted in dose-dependent reduction of cell growth in *KMT2A-r* cell lines with a rather subtle increase in dead cells (Fig. 2A), while leaving non-*KMT2A-r* cell lines largely unaffected. The only non-KMT2A-r cell line responding in a dose-dependent manner was the *NPM1*-mutated OCI-AML3 line with dependency on a shared oncogenic gene expression program. Cell cycle progression was also affected by pharmacologic inactivation of PSMB8 in KMT2A-r cell lines (Supplemental Figure S2A-B).

To confirm this selective vulnerability on primary human AML in vivo, we transplanted patient-derived xenografts (PDXs) harboring an KMT2A::MLLT3 (MLL-AF9) or KMT2A::AFF1 (MLL-AF4) fusion, into NOD.Cg^KitW−41J^Prkdc^scid^Il2rg^tm1Wjl^ (NSGW41) animals. The relative abundance of human AML cells in the recipients’ peripheral blood was measured over time by flow cytometry, and PR-957 treatment was initiated once hCD45^+^ AML cells constituted 0.05%–0.9% of peripheral blood (PB) cells (Supplementary Figure S2C). PR-957 treatment was initiated at 3 mg/kg i.v. for 5 consecutive days and continued every 2 weeks with dose escalation to 6 mg/kg. While *KMT2A-r* leukemic cells engrafted at equal numbers, they failed to expand and outcompete murine hematopoiesis when exposed to PR-957, resulting in improved survival of animals receiving KMT2A::MLLT3 (*p* = 0.0004) or KMT2A::AFF1 (*p* < 0.0001) xenograft cells (Fig. 2B, Supplementary Figure S2D). Flow cytometric analysis of bone marrow (BM) and spleen compartments showed immunophenotypic eradication of leukemic cells in 2/7 animals transplanted with KMT2A::MLLT3 and 6/8 animals transplanted with KMT2A::AFF1 leukemia after PR-957 treatment (Supplementary Figure S2E). Together, these results suggest a critical requirement of PSMB8 function for proliferation and clonogenicity of human *KMT2A-r* AML cells.

### KMT2A-r leukemia initiation depends on PSMB8/LMP7

**Fig. 3:**
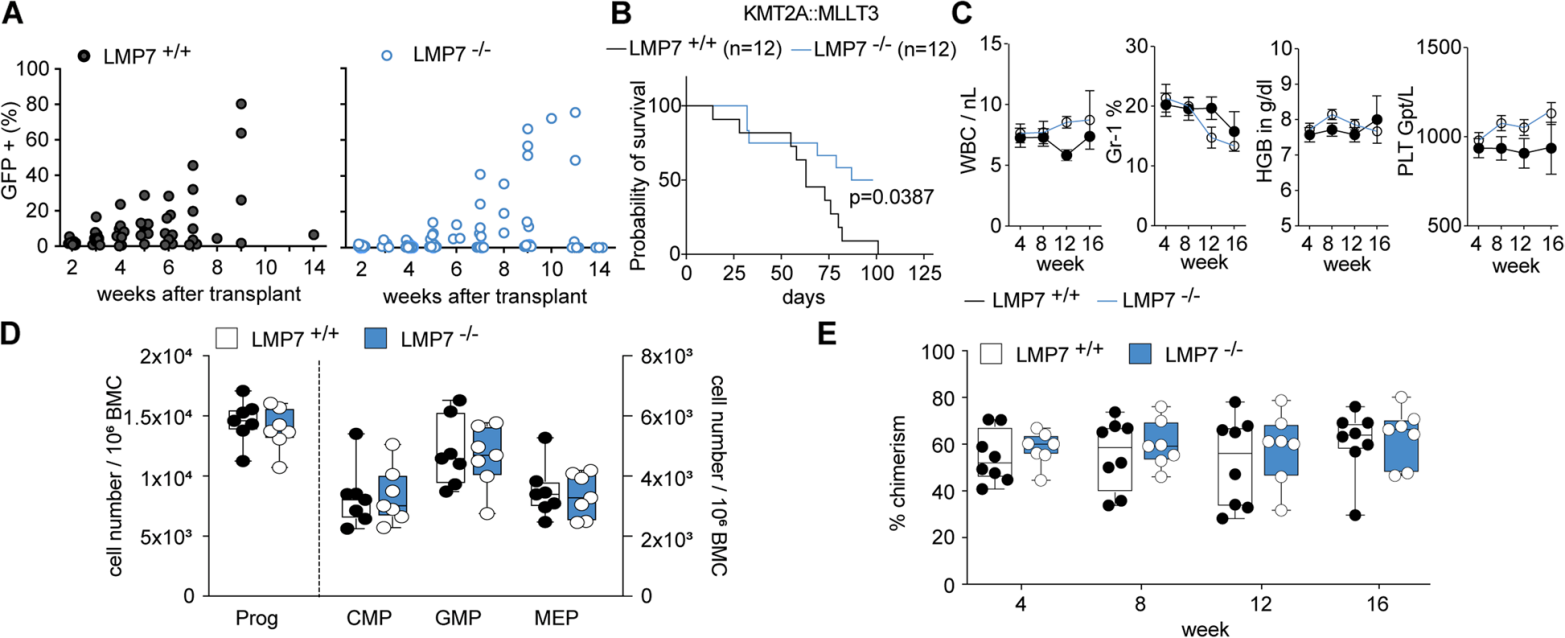
LMP7/PSMB8 is essential for KMT2A-r AML development but dispensable for normal hematopoiesis. **A** Dot plots depicting % of GFP^+^ cells in peripheral blood of recipient mice transplanted with 7 × 10^4^ KMT2A::MLLT3 transformed LSKs from LMP7^+/+^ (*n *= 12) or LMP7^−/−^ (*n* = 12) mice over 14 weeks. Two independent cohorts. **B** Kaplan–Meier survival curves of recipient mice (KMT2A::MLLT3 transformed LSKs from LMP7^+/+^ (*n* = 12) or LMP7 ^−/−^ (*n* = 12) mice). Two independent cohorts; Mantel-Cox test. **C** White blood counts (WBC), hemoglobin (HGB) and platelets (PLT) in the peripheral blood of LMP7^−/−^ (*n* = 7) for 16 weeks of steady-state hematopoiesis, compared with LMP7^+/+^ controls (*n* = 7). **D** Immunophenotypic quantification of progenitor cell abundance (Prog: Lin^−^ Sca1^−^ c-Kit^+^), common myeloid progenitors (CMP: Lin^−^ Sca1^−^ c-Kit^+^ CD34^+^ FcgR^−^), granulocyte–macrophage progenitors (GMP: Lin^−^ Sca1^−^ c-Kit^+^ CD34^+^ FcgR^+^) and megakaryocyte-erythroid progenitors (MEP: Lin^−^ Sca1^−^ c-Kit^+^ CD34^−^ FcgR^−^) in the bone marrow. **E** Peripheral blood chimerism over 16 weeks; competitive repopulation assay using BM cells from LMP7^−/−^ (*n* = 8) or LMP7^+/+^ (*n* = 8) donors

To assess for the requirement of immunoproteasome function for leukemia initiation, we used a retroviral model of leukemic transformation. Transformation of murine stem and progenitor cells with oncogenic KMT2A-fusions results in aberrant self-renewal, unlimited re-plating capacity in methylcellulose and rapid leukemia onset in vivo following transplantation into irradiated recipient hosts [22, 23]. We used murine Lineage^−^ Sca1^+^ c-Kit^+^ (LSK) progenitor cells derived from a conventional PSMB8 (murine: LMP7) knockout mouse model [24], where exons 1–5, encoding the first 247 amino acids of the protein, are genetically deleted and lead to loss of a functional protein. Cells were isolated from the bone marrow (BM) of the respective donor animals as published before [25, 26]. LMP7^+/+^ or LMP7^−/−^ LSK cells were then transduced with KMT2A-oncogenes (KMT2A::MLLT3 or KMT2A::MLLT1) followed by serial re-plating in methylcellulose to assess colony forming capacity and self-renewal capacity in vitro. While LMP7^+/+^ cells showed unlimited re-plating capacity, LMP7^−/−^ cells failed to sustain colony growth beyond 3 rounds of serial re-plating (Supplementary Figure S3A). To investigate whether immunoproteasome function is required for leukemia development in vivo, LMP7^+/+^ and LMP7^−/−^ LSK cells were transduced with the KMT2A::MLLT3oncogene (MSCV-KMT2A::MLLT3–GFP) and a total of 7 × 10^4^ transduced (GFP^+^) cells were injected into sublethally (7 Gy) irradiated recipient hosts (Supplementary Figure S3B). When followed over time, recipients of LMP7-deficient cells showed delayed onset of leukocytosis and delayed increase of GFP^+^ cells in the PB (Fig. 3A). This delay in disease development resulted in significantly prolonged survival (median survival of MA9-LMP7^+/+^: 63.0 days; MA9-LMP7^−/−^: 92.5 days; *p* = 0.0387) and 6/12 (50%) of animals failed to establish leukemia within 100 days of observation (Fig. 3B). To validate the requirement of immunoproteasome function for leukemia development, we used a Doxycycline (DOX) inducible mouse model of KMT2A:: MLLT1 (i- KMT2A::MLLT1). BM cells of i- KMT2A::MLLT1 (treated for 2 weeks with DOX) were transduced with two independent shRNAs against LMP7 (sh1- and sh4-LMP7) or a non-targeting control (shNT). 1 × 10^6^ transduced cells were transplanted into CD45.1 recipient mice (kept in food supplement with 0.545 g/kg of DOX) (Supplementary Figure S3C). Recipients of LMP7-deficient cells had a delay in disease development and significant prolonged survival (median survival of sh1-LMP7 i-KMT2A::MLLT1: not reached, *p* = 0.1108; sh4-LMP7 i-KMT2A::MLLT1: 123 days, *p* = 0.0029; shNT i-KMT2A::MLLT1: 71 days) (Supplementary Figure S3D-E). These findings indicate a requirement of LMP7 for development and propagation of KMT2A-r induced leukemia in vitro and in vivo. To assess for the role of LMP7 in non-leukemic, normal HSPC function, we monitored peripheral blood (PB) counts and distribution of immune cell subsets of LMP7 knockout mice compared to wildtype littermate controls over 4 months but failed to detect abnormalities in white blood count, hemoglobin or platelet count (Fig. 3C). Consistently, immunophenotypic analysis of BM at 4 months of age revealed no significant quantitative changes in all HSPC subsets (Fig. 3D). To dissect the functional influence of LMP7 deletion on long-term (LT)-HSC versus more differentiated progenitors (short-term (ST)-HSC/multipotent progenitors—MPP), we investigated short-term colony formation in vivo. Colony forming unit spleen cells repopulate irradiated recipients for 1–3 weeks but fail long-term engraftment [27]. Injection of 100 sorted LMP7^−/−^ LSK cells resulted in pronounced spleen colony formation (Supplementary Figure S3F), which was comparable to the colony numbers generated by LMP7^+/+^ littermate controls. These findings indicate that LMP7 is dispensable for short-term repopulation and multipotent progenitor function. To test for the function of LMP7 deficient HSC, we performed competitive transplantation into irradiated recipient hosts (Supplementary Figure S3G). When LMP7^−/−^ cells or LMP7^+/+^ controls of 6–8 week old donor mice were transplanted into primary recipient hosts in a competitive manner at a ratio of 1:1 we found no loss of function in LMP7 deficient cells as they competed against wildtype controls as indicated by stable PB chimerism over 16 weeks (Fig. 3E). Taken together, inactivation of LMP7 does not impair normal HSCP function in vivo.

### Pharmacologic inhibition of PSMB8/LMP7 impairs murine KMT2A-r leukemia stem cell (LSC) self-renewal

**Fig. 4:**
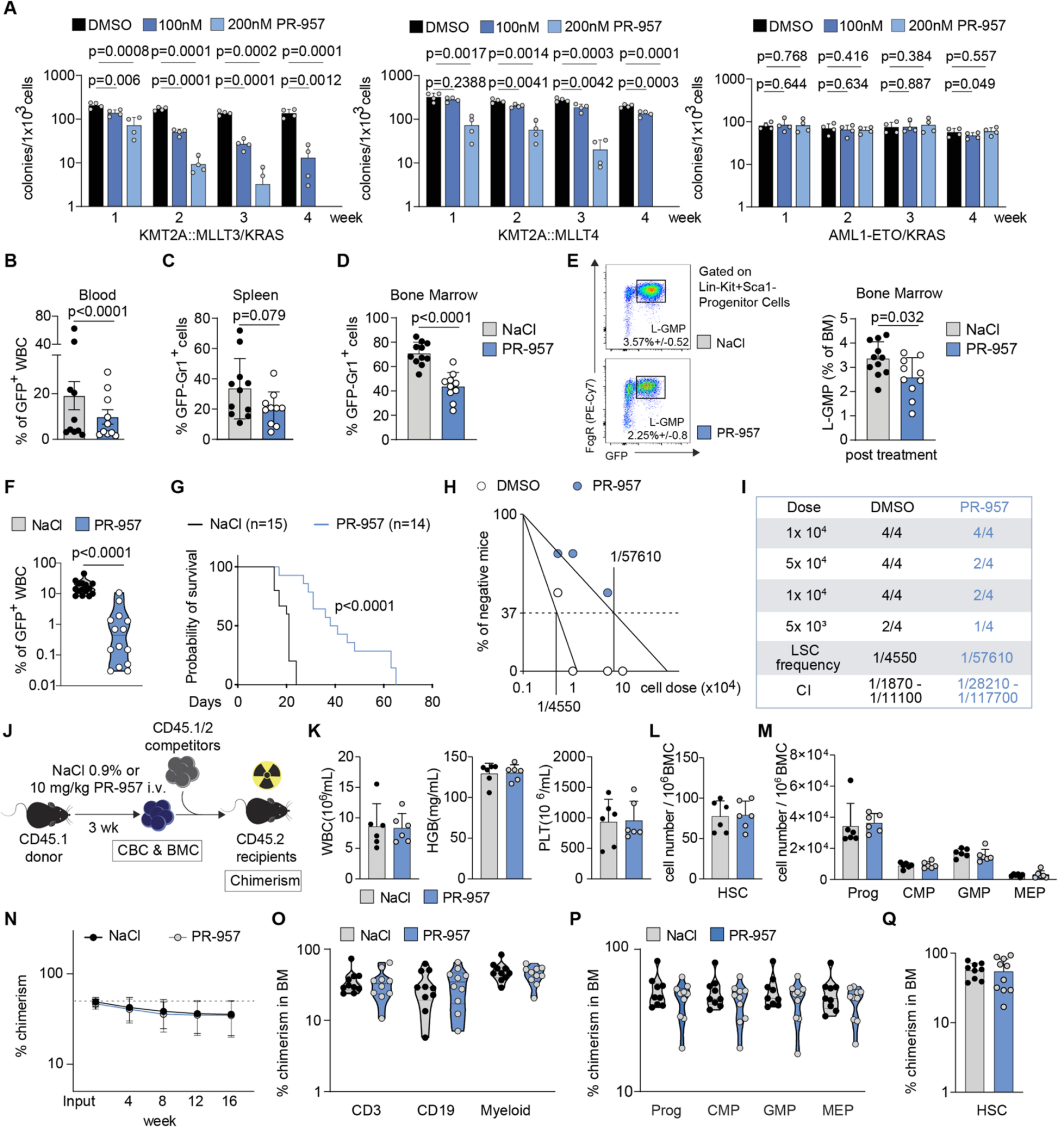
Pharmacologic inhibition of LMP7/PSMB8 impairs KMT2A-r leukemia stem cell function without affecting normal hematopoietic stem cells. **A** Serial re-plating to assess for colony formation in methylcellulose using murine LSK cells transformed with KMT2A::MLLT3/KRAS, KMT2A::MLLT4 or AML1-ETO/KRAS. Cells were treated with DMSO as diluent control or PR-957 (100 nM, 200 nM). *n* = 3–4 independent experiments; mean with SD; paired Student t test. **B-D** 3 × 10^5^ KMT2A::MLLT3 murine BM cells transplanted into sublethally irradiated recipient mice. Mice were treated for 5 days/week for 3 cycles with 10 mg/kg PR-957 (*n* = 10) or NaCl 0.9% as diluent control (*n* = 11). Relative abundance of leukemic cells after 3 weeks of treatment in primary recipient mice in (**B**) peripheral blood, (**C**) spleen and (**D**) bone marrow. Two independent cohorts; mean with SD; Mann–Whitney U test. **E** Relative abundance of L-GMP (Lin-Kit + Sca1-CD34 + FcgR + GFP +) in diluent or PR-957 treated mice. Two independent cohorts; mean with SD; Mann–Whitney U test. **F** 2 × 10^6^ whole bone marrow cells from the PR-957 or NaCl in vivo treated KMT2A::MLLT3 mice were transplanted into secondary recipients (*n* = 14 recipients of PR-957 treated mice; *n* = 15 recipients of NaCl treated mice). Abundance of leukemic cells in the peripheral blood, 2 weeks after transplantation. Two independent cohorts; Mann–Whitney U test. **G** Survival of secondary recipient mice. Mantel-Cox test. **H-I** Limiting dilution (LD) assay using murine KMT2A::MLLT3 BM cells treated for 48 h with diluent or 200 nM PR-957. **H** Reduction of leukemia initiating cells and (**I**) Cell dose, animal numbers, LSC-frequency and confidence intervals (CI) following diluent or PR-957 exposure. *n* = 4 mice per dilution and treatment, analysis performed using ELDA (Extreme Limiting Dilution Assay) software [28]. **J** Schematic depicting competitive repopulation assay to investigate effects of PR-957 treatment on normal HSPCs. **K-M** Relative abundance of hematopoietic cells in CD45.1 mice after 3 weeks of treatment with 10 mg/kg PR-957 (*n* = 6) or NaCl 0.9% (*n* = 6), specifically in (**K**) Peripheral blood, (**L-M**) Bone marrow. **L** HSC abundance (HSC: Lin^−^ Sca1^+^ cKit^+^ CD48^−^ CD150^+^); (**M**) Progenitor cell abundance; (**N**) Peripheral blood chimerism of recipient animals using BM cells from in vivo treated mice with PR-957 or diluent control. **O-Q** Abundance of hematopoietic cells in the BM of recipient mice at week 16. **O** mature cell compartments; (**P**) progenitor cells; (**Q**) HSCs (HSC: Lin^−^ Sca1^+^ cKit^+^ CD34^−^)

Gene expression programs induced by KMT2A-fusion proteins confer stemness and aberrant self-renewal capacity to committed progenitors [1]. To study the effects of *PSMB8* (murine: LMP7) inactivation on LSC self-renewal, we used established models of murine, KMT2A-fusion driven leukemia. Exposure of KMT2A::MLLT3/KRAS or KMT2A::MLLT4 induced murine leukemic cells to PR-957 at nanomolar concentrations resulted in profound dose dependent reduction of clonogenic potential, which was not detectable when treating non-KMT2A fusion oncogenes such as AML1-ETO/KRAS (Fig. 4A). Moreover, treatment with PR-957 resulted in reduction of colony size and changes in colony shape, specifically in *KMT2A-r* leukemia (Supplementary Figure S4A).

As leukemic cells are highly heterogeneous, we also aimed to assess the LSC pool that frequently persists after treatment and is a source of clinical relapse. Murine HSPCs were transduced with KMT2A::MLLT3 (MA9) to induce leukemia in sublethally irradiated primary recipient mice (Supplementary Figure S4B). Primary recipients were treated with 10 mg/kg PR-957 i.v. QD for 3 weeks (5 days/week) and PB and BM were investigated 3 days after treatment discontinuation. Leukemic cells in the PB, spleen, lung and liver of PR-957 treated animals were reduced compared to diluent treated controls (Fig. 4B-C; Supplementary Figure S4C). Consistently, we found significant decrease in the abundance of leukemic cells in the BM of PR-957 treated mice (42.9% versus 69.8%; *p* < 0.0001; Fig. 4D). Next, we assessed the frequency of L-GMPs (Lin^−^ Sca1^−^ c-Kit^+^ CD34^+^ FcgR^+^) by flow cytometry, which have been described as the functionally relevant LSC population in MA9 driven leukemia [29]. Of note, PR-957 treated animals showed significant reduction of L-GMPs compared to diluent treated controls (2.25 ± 0.8% and 3.57 ± 0.52%, respectively, *p* = 0.032; Fig. 4E). Injection of 1 × 10^6^ total BM cells into sublethally irradiated secondary recipient mice resulted in decreased numbers of leukemic cells in the peripheral blood two weeks after transplantation (17.2 vs. 1.6 Gpt/L GFP^+^ WBC; *p* < 0.0001; Fig. 4F) and significant delay in disease development for recipients of PR-957 treated cells (median survival 39.5 vs. 21 days; *p* < 0.0001; Fig. 4G). To determine the functional abundance of LSCs and the leukemia initiating potential, we performed limiting dilution assays by injecting sublethally irradiated recipient mice (7 Gy) with limiting numbers (1 × 10^5^, 5 × 10^4^, 1 × 10^4^, 5 × 10^3^) of GFP^+^ leukemic BM cells. PR-957 treated cells showed profound, more than log-fold reduction in LSC frequency compared to diluent treated controls (1/57610, CI 1/1870–1/11100 versus 1/4550, CI 1/28210–1/117700; Fig. 4H-I). Taken together, pharmacologic immunoproteasome inhibition using the specific PSMB8/LMP7 inhibitor PR-957 attenuated the leukemia initiating potential of *KMT2A-r* AML cells in vivo*.* This effect was even more pronounced in the LSC (L-GMP) population, indicating reduced fitness and decreased LSC numbers as potential causes. To examine for potential toxicity to normal hematopoietic stem- and progenitor cells (HSPCs), 6–8 week old C57BL/6 animals were treated with 10 mg/kg PR-957 i.v. QD for 3 weeks (5 days/week) (Fig. 4J). In contrast to our findings in *KMT2A-r* leukemic cells PSMB8/LMP7 function appeared to be dispensable for steady state hematopoiesis regarding PB counts (Fig. 4K) and immunophenotypic abundance of BM HSPCs (Fig. 4L-M). Likewise, normal murine HSC function was not impaired during competitive transplantation (Fig. 4J) as indicated by PB chimerism of primary recipient hosts (Fig. 4N) and abundance of BM HSPCs at week 16 (Fig. 4O-Q, Supplementary Figure S4D).

### PSMB8 inhibition increases BASP1 which represses KMT2A-fusion target gene expression

**Fig. 5:**
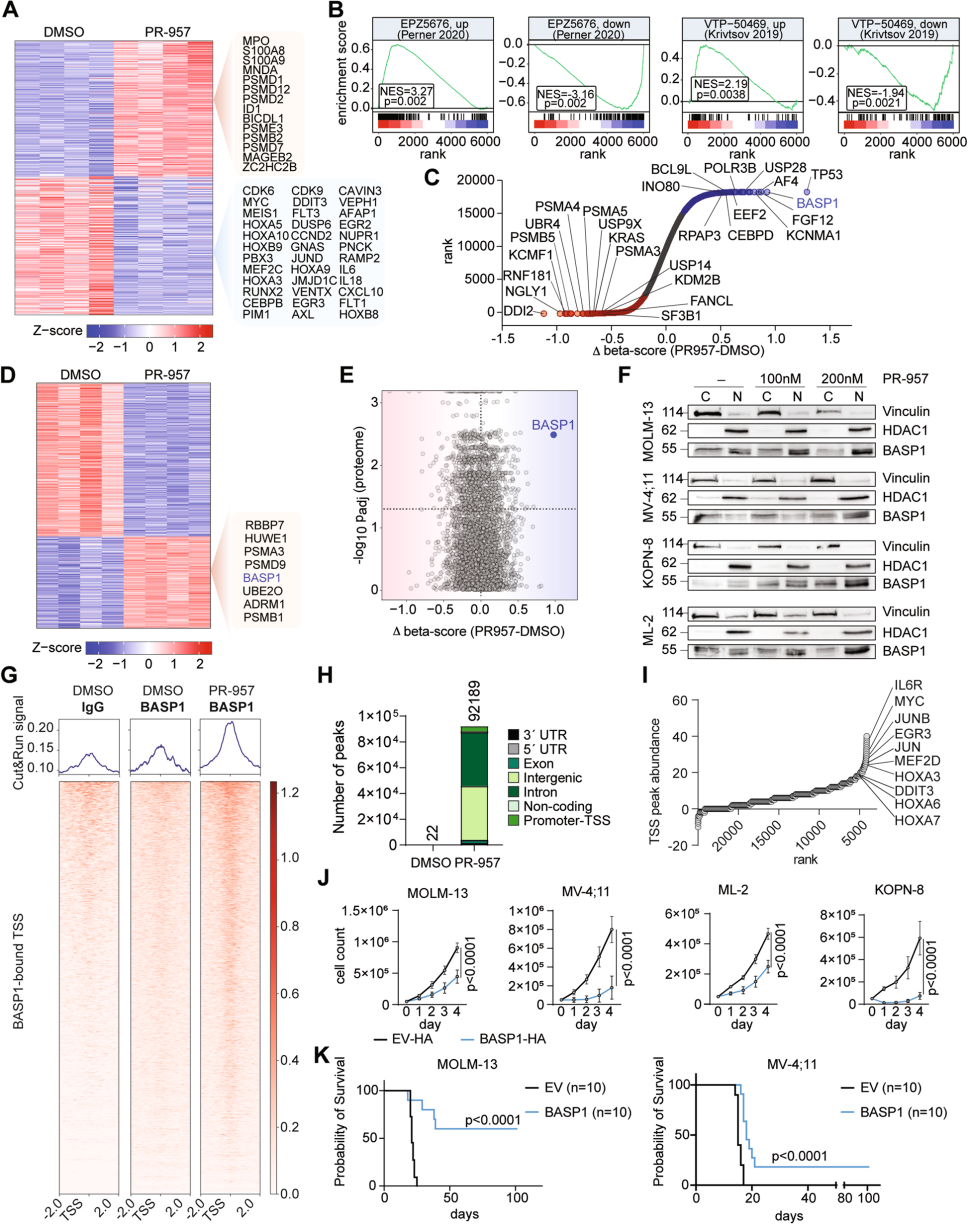
Enrichment of BASP1 by PSMB8 inhibition inhibits KMT2A target gene expression. **A** Heatmap of differentially expressed genes in MOLM-13 cells: 100 nM PR-957 vs. DMSO (72 h). Upregulated (red; fold-change (FC) > 2, *p* < 0.05) and downregulated (blue; FC < -2, *p* < 0.05) genes. **B** Gene Set Enrichment Analysis (GSEA) of PR-957 treated MOLM-13 cells compared to EPZ5676- or VTP-50469-treatment; NES (normalized enrichment score). **C** Hockey-stick-plot of ranked genes found to be differentially enriched/depleted in genome wide CRISPR/Cas9 screening in MOLM-13 cells. **D** Heatmap depicting unsupervised hierarchical clustering of differentially abundant proteins detected by mass-spectrometry. 100 nM PR-957 vs. DMSO, 72 h, MOLM-13. Upregulated (red; FC > 2, *p* < 0.05) and downregulated (blue; FC < -2, *p* < 0.05) proteins. **E** Correlation between the magnitude of differential protein abundance (-log10 Padj) as determined by proteome analysis and the functional dependencies obtained by CRISPR/Cas9 screening (Δ beta-scores PR-957 vs. DMSO) in MOLM-13 cells. **F** Western Blotting showing expression of BASP1 in nuclear (N) and cytoplasmic (C) fractions of MOLM-13, KOPN-8, ML-2 and MV4;11 cells. PR-957 (100 nM, 200 nM) vs. DMSO, 72 h. **G** Heatmaps displaying IgG and BASP1 Cut&Run signal mapping to a 2-kb window around the TSS (Transcription Start Site). 100 nM PR-957 vs. DMSO, 48 h, MOLM-13 cells; 1 (out of *n* = 3) representative replicate. **H** Stacked bar plot depicting genomic distribution of BASP1 Cut&Run peaks. MOLM-13 cells; *n* = 3 independent replicates. **I** Dot-plot of ranked BASP1-bound TSS according to their abundance; 1 representative replicate. **J** Growth curves of KMT2A-r cells (MOLM-13, MV-4;11, ML-2, KOPN-8) transduced with pLEX-BASP1-HA-Tag (BASP1-HA) or pLEX-HA-Tag (EV-HA). *n* = 4 independent experiments, in triplicate; mean with SEM; 2-way ANOVA. **K** Survival curves of NXG recipient mice transplanted with 1 × 10^5^ MOLM-13 or MV-4;11 cells expressing pLEX-BASP1 (BASP1) or pLEX-EV (EV) (*n* = 10 per construct and cell line). Two independent cohorts; Mantel-Cox test

To determine to what extent the inhibition of PSMB8 modulates oncogenic gene expression programs, we sought to investigate transcriptional regulation in *KMT2A-r* cells. Global transcriptome analysis by RNA-sequencing performed in MOLM-13 cells revealed differential regulation of 973 down- and 991 upregulated genes upon PR-957 treatment (Supplementary Figure S5A). Among the significantly induced genes we found compensatory up-regulation of proteasome subunits (Fig. 5A). Unexpectedly, down-regulated genes included several bona fide KMT2A-target genes such as *HOX*-genes (*HOXA*-cluster, *PBX3*, *MEIS1*, *VENTX*), cell cycle regulators (*CDK6*, *CDK9*), transcription factors (*MEF2C*, *RUNX2*) and signaling molecules (*FLT3*, *AXL*, *GNAS*), among others (Fig. 5A). Consistently, genes deregulated by epigenetic inhibitors of the KMT2A-complex (Dot1L- or Menin-inhibitors) were enriched in the gene sets regulated by pharmacologic PSMB8 inhibition (Fig. 5B). To assess for changes on chromatin as consequence of PSMB8 inhibition, we performed Cut&Tag-sequencing for KMT2A-related histone tail modifications and ATAC-sequencing to assess for early changes in chromatin conformation (Supplementary Figure S5B-E).

Of note, neither changes in KMT2A-complex associated histone tail modifications nor changes in chromatin accessibility could be detected. These findings suggest an inhibitory effect of PSMB8 inactivation on expression of KMT2A-target genes that is not mediated by modulation of epigenetic complexes, including KMT2A-function. To identify functional effectors of PSMB8 activity, we applied a genome-wide CRISPR/Cas9 screen in the human KMT2A-r cell line MOLM-13. This cell line was selected for its sensitivity to PR-957 (Fig. 2A) and ability to be efficiently transduced among the KMT2A-r cell lines evaluated above. For the screen, MOLM-13 cells were infected with the human Liu lentiviral library, containing 92,817 single guide RNAs targeting against 18436 genes and treated with PR-957 (or DMSO, as control) for 12 days (Supplementary Figure S5F-G). Use of the MAGeCK-MLE and FluteMLE algorithms [30] offered the opportunity to specifically identify synthetic lethal hits and resistance mediators, as well as genes that are potential downstream effectors of the drug target. While deletion of molecules functionally related to the ubiquitin–proteasome system (UPS) could be identified as sensitizers to PR-957 treatment, several transcriptional regulators, such as CEBPD, RPAP3, POLR3B, AF4, BASP1 and TP53 (Fig. 5C) scored as resistance mediators. To assess whether disrupting homeostasis of transcriptional regulators by immunoproteasome inhibition could account for the observed repression of KMT2A-target genes, we performed in-depth proteome analysis on MOLM-13 cells with or without exposure to PR-957 (Fig. 5D). Overall, 1329 proteins (500 up and 829 down) showed differential abundance in response to PR-957 treatment. Consistent with transcriptomic changes, compensatory upregulation of UPS-related proteins was detectable. Among the transcriptional regulators classified as resistance mediators by functional CRISPR/Cas9 screening, protein abundance of BASP1 was significantly increased in global proteome analysis (Figs. 5D-E). As also seen by Western Blotting, abundance of BASP1 protein levels increased in a dose dependent manner and specifically in the nuclear fraction of PR-957 (Fig. 5F) as well as Bortezomib (BTZ) and Carfilzomib (CFZ) (Supplementary Figure S5H-I) treated MOLM-13, MV-4;11, KOPN-8 and ML-2 cells. Therefore, we sought to confirm DNA-binding of BASP1 by Cut&Run sequencing. Here, we found that pharmacologic inhibition of PSMB8 by PR-957 treatment increased BASP1 binding, including at active transcriptional start sites (TSS) (Fig. 5G-H). Notably, specifically TSS of KMT2A-target genes appeared occupied by BASP1 upon PSMB8-inhibition (Figs. 5I, Supplementary Figure S5J). To assess for the functional consequences of increased BASP1 expression and to confirm its repressive role, we conducted (lentiviral and CRISPRa-induced) overexpression studies in KMT2A-r cell lines. Increased abundance of BASP1 attenuated growth of KMT2A-r AML cells in vitro and reduced leukemia formation in humanized recipient mice (Fig. 5J-K, Supplementary Figure S6). Together, these findings indicate a functional role of BASP1 as a transcriptional repressor of KMT2A-target genes that may be independent from the epigenetic functions of the oncogenic KMT2A-fusion protein.

### Combined targeting of oncogenic gene expression through pharmacologic inactivation of Menin and PSMB8

**Fig. 6:**
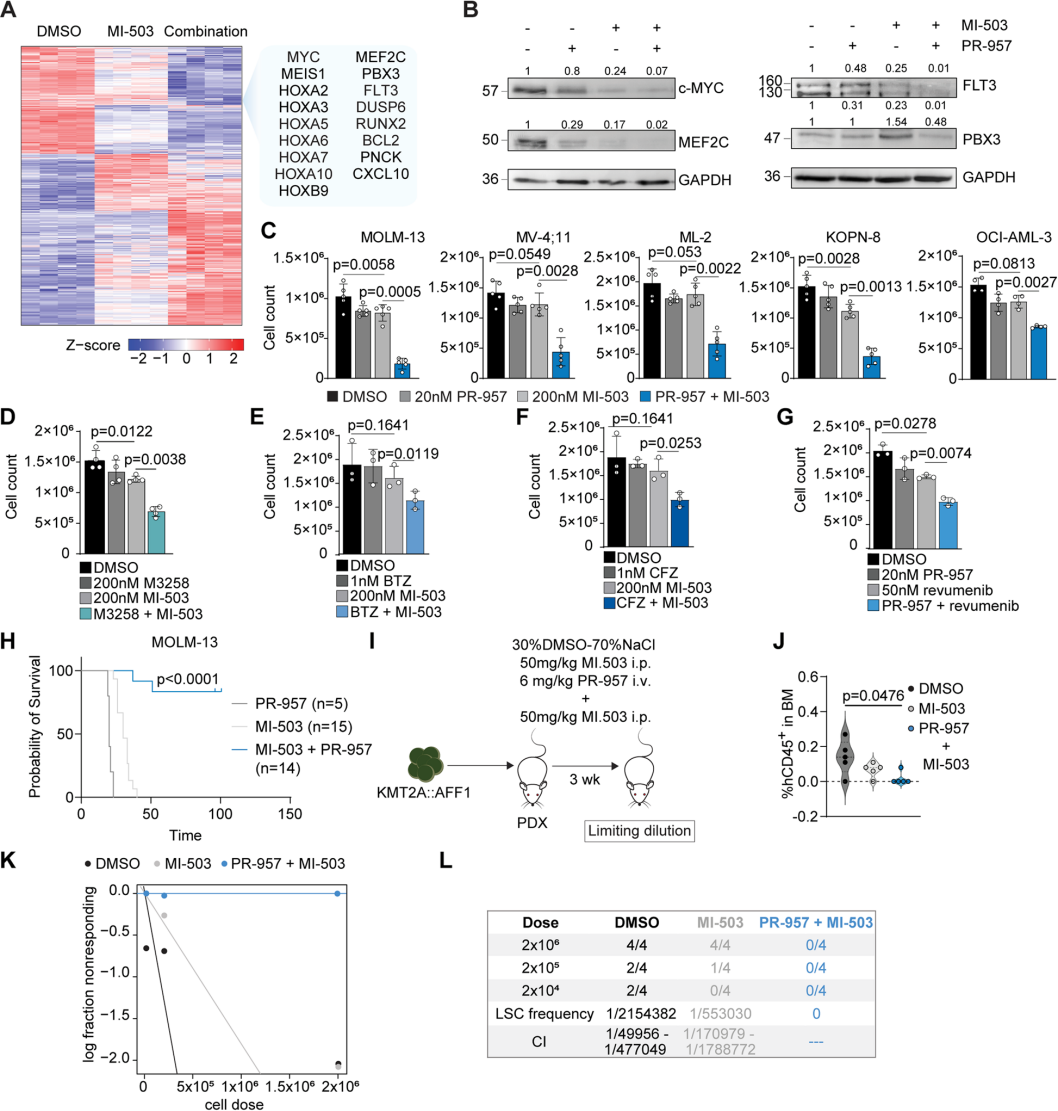
Synergistic targeting of oncogenic gene expression through pharmacologic inactivation of Menin and PSMB8. **A** Heatmap of differentially expressed genes in MOLM-13 cells. 1 μM MI-503 treatment, 1 μM MI-503 and 100 nM PR-957 treatment or DMSO for 72 h. Upregulated (red; FC > 2, *p* < 0.05) and downregulated (blue; FC < -2, *p* < 0.05) genes. **B** Representative plots (out of *n* = 3–4) showing protein expression of c-Myc, MEF2C, FLT3 and PBX3 upon treatment with DMSO, 100 nM PR-957, 1 μM MI-503 or 1 μM MI-503 + 100 nM PR-957 for 72 h in MOLM-13 cells. **C** Bar plots depicting cell counts in MOLM-13, MV-4;11, ML-2, KOPN-8 and OCI-AML3 cells after treatment with 20 nM PR-957, 200 nM MI-503, a combination of both or DMSO for 6 days. *n* = 4–5 independent experiments; mean with SD; paired Student t test. **D-G** Bar plots representing proliferation of MOLM-13 cells after treatment with indicated monotherapies, combinations or DMSO for 6 days. *n* = 3 independent experiments; mean with SD; paired Student t test. **H** Xenograft of human MOLM-13 cells: Survival curve of NXG recipient mice transplanted with 1 × 10^5^ MOLM-13 cells pre-treated ex vivo with either 100 nm PR-957 (*n* = 5) for 48 h, 2.5 μM MI-503 (*n* = 15) for 96 h or a combination of 2.5 μM MI-503 for 96 h and 100 nM PR-957 for 48 h (*n* = 14). Four independent cohorts; Mantel-Cox test. **I** Patient derived xenograft (PDX): Schematic representation of the in vivo MI-503 (50 mg/kg, × 7), MI-503 + PR-957 (6 mg/kg, i.v., 5 days/week for 3 weeks) or 30% DMSO-70% NaCl0.9% (× 7) treatment of NXG mice (*n* = 5/treatment) injected with 2 × 10^4^ KMT2A::AFF1 PDX-cells. BM cells were subsequently transplanted at limiting numbers (2 × 10^6^, 2 × 10^5^, 2 × 10^4^) into NXG recipient mice. **J** Immunophenotyping of human CD45^+^ (hCD45^+^) cells in the BM of NXG mice after in vivo treatment with MI-503, MI-503 + PR-957 or DMSO/NaCl0.9%. *n* = 5 mice per treatment; Mann–Whitney U test. **K-L** Limiting dilution (LD) assay. **K** Reduction of leukemia initiating cells. **L** Cell dose, animal numbers, LSC-frequency and CI following diluent, MI-503 or PR-957 + MI-503 exposure. *n*= 4 per dilution and treatment; analysis was performed using ELDA software [28]

These results prompted us to assess for enhanced efficacy when combining inhibitors of the KMT2A-complex with immunoproteasome inhibitors. Combinatorial use of Menin-inhibitor MI-503 (1 μM, 72 h) with immunoproteasome inhibitor PR-957 at nanomolar concentrations (100 nM, 72 h) resulted in enhanced repression of the KMT2A-related gene expression program (Fig. 6A).

Consistently, abundance of relevant transcriptional effectors such as MEF2C, FLT3 or PBX3 (and partially c-MYC) was more efficiently reduced by combined targeting compared to monotherapy (Fig. 6B, Supplementary Figure S7A). Combinatorial treatment efficiently attenuated proliferative capacity of KMT2A-r or NPM1-mutated cell lines in vitro (Fig. 6C) without induction of apoptosis (Supplementary Figure S7B). These findings could be recapitulated when using other specific (M3258; Fig. 6D) or non-specific (Bortezomib, BTZ; Carfilzomib, CFZ; Figs. 6E-F) inhibitors of the immunoproteasome or second-generation clinical grade Menin-inhibitors (Revumenib (SNDX-5613); Fig. 6G), confirming a class-effect.

**Fig. 7:**
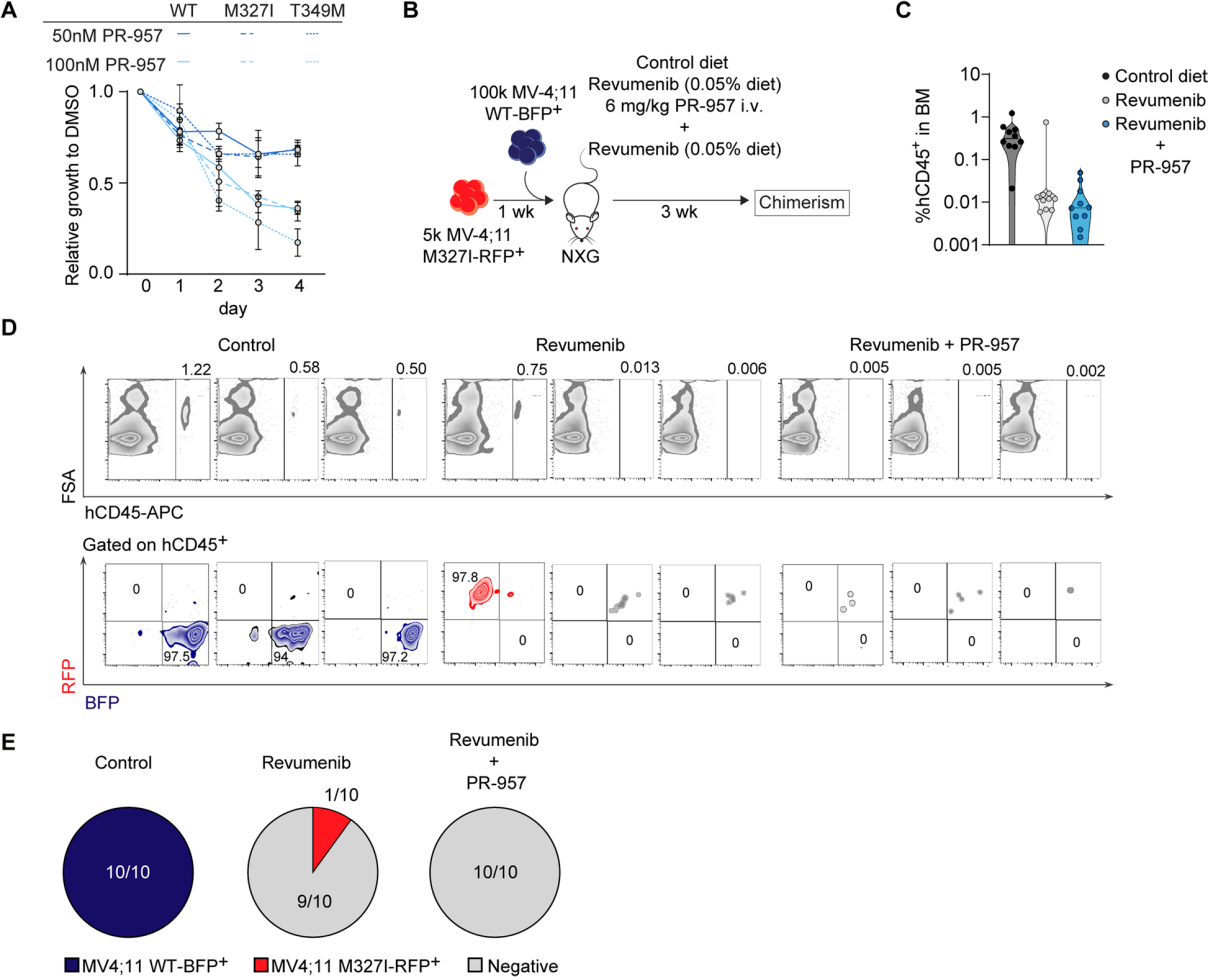
Menin mutated cells with acquired resistance to Menin-inhibition retain sensitivity to immunoproteasome inhibition and preserved activity of combinatorial treatment strategies against MEN1-mutated clones may blunt outgrowth of resistant cells. **A** Relative growth to DMSO of wild-type MV-4;11 (MV-4;11 WT) and two MV-4;11 cell lines containing mutations in MEN1 (MV-4;11 M327I, methionine to isoleucine change at position 327; MV-4;11 T349M, threonine to methionine at position 349) after treatment with PR-957 (50 nM, 100 nM). *n* = 4 independent experiments; mean with SD. **B** Schematic representation of the competitive transplantation of 5 × 10^4^ MV-4;11 M327I-RFP^+^ cells and 1 × 10^5^ MV-4;11 WT-BFP^+^ cells (transplanted one week later) into NXG mice. Recipient mice were treated for 3 weeks with food supplemented with 0.05% Revumenib, Revumenib + PR-957 (6 mg/kg, 5 days/week, at week 1 and week 3) or control diet. **C** Immunophenotyping of human CD45 + (hCD45 +) cells in the BM of NXG mice after in vivo treatment with Revumenib, Revumenib + PR-957 or control diet. *n* = 10 mice per treatment. **D** Representative flow cytometry plots (3 per cohort) showing the percentage of hCD45 + and percentage of RFP + hCD45 + and BFP + hCD45 + bone marrow cells in the control, Revumenib-treated and Revumenib + PR-957 treated mice. **E** Pie charts depicting the number of mice expressing MV-4;11 WT-BFP + cells (blue), MV-4;11 M327I-RFP + cells (red) or no RFP + / BFP + cells (negative, grey), respectively

Combinatorial treatment of MI-503 (2.5 μM, 96 h) with PR-957 (100 nM, 48 h) in MOLM-13 cells resulted in prolonged survival of recipient NXG mice as compared to MI-503 and PR-957 monotherapy (MI-503: 30 days; PR-957: 20 vs. comb: not reached; *p* < 0.0001) and reduced disease penetrance by 83.3% (Fig. 6H). We sought to validate these findings by in vivo treatment of patient derived xenograft (PDX) models and assess for quantitative reduction of leukemia initiating cells by limiting dilution assays (Fig. 6I). Assessment of leukemic bone marrow infiltration in treated primary recipients revealed pronounced reduction of human CD45^+^ leukemic cells (MI-503: median 57%, Combination: 100%) compared to diluent control (DMSO/NaCl0.9%; Fig. 6J). When injected into secondary recipient mice at limiting numbers (2 × 10^6^, 2 × 10^5^, 2 × 10^4^) recipients of MI-503 + PR957 treated bone marrow cells showed profound reduction in LSC frequency compared to MI-503 or diluent treated controls (0 versus 1/553030, CI 1/170979–1/1788772; 0 versus 1/2154382, CI 1/49956–1/477049, respectively; Fig. 6K-L). Moreover, combinatorial treatment prolonged survival (median survival not reached) compared to MI-503 (median survival 2 × 10^6^:114.5 days; for 2 × 10^5^: not reached, 2 × 10^4^: not reached; *p* < 0.0001) or diluent controls (median survival 2 × 10^6^: 114 days; 2 × 10^5^: 225 days; 2 × 10^4^: 211.5 days; *p* < 0.0001) and reduced hCD45 + leukemic cells in recipient BM (Supplementary Figure S7C-D). Recently, Menin-inhibitors have shown promising clinical responses in patients with relapsed/refractory KMT2A-r or NPM1c AML [31]. However, more than one third of patients on prolonged inhibitor treatment acquired resistance to Menin-inhibition through somatic mutations in *MEN1 *[32]. As these findings indicate evolution of escape mutants upon chromatin-complex-inhibitors, we sought to assess for the efficacy of immunoproteasome inhibition in the context of resistance mediating mutations. KMT2A-r cell lines MV-4;11 harboring the M327I- and T349M-resistance mutations were exposed to increasing concentrations of PR-957 (50 nM, 100 nM). Notably, immunoproteasome inhibition efficiently attenuated cell growth in MV-4;11-WT and MV-4;11-M327I or MV-4;11-T349M mutants to a similar extent, indicating preserved sensitivity (Fig. 7A) while both cell lines showed preserved resistance against Menin-inhibitors (MI-503 and Revumenib; Supplementary Figure S8). To assess for the effects of combined targeting on cell competition in vivo, we injected MV-4;11 wildtype (-BFP) and MV-4;11-M327I (-RFP) in a 20:1 ratio sequentially into immunocompromised NXG animals and treated them over 3 weeks either with diluent/chow control, 0.05% diet of the Menin-inhibitor Revumenib or a combination of Revumenib with 6 mg/KG PR-957 i.v. (overlapping for 2 out of 3 weeks) (Fig. 7B). As previously published, treatment with Revumenib efficiently eradicates KMT2A-r MV-4;11-WT cells in NXG mice, while there was rapid leukemia development in diluent/control chow treated controls (Fig. 7C). Outgrowth of MV-4;11-M327I resistant clones could be observed in 10% of recipient animals upon Revumenib monotherapy. In contrast, combination of Revumenib with PR-957 resulted in complete eradication of human CD45 + leukemic cells and prevented outgrowth of Menin-inhibitor resistant mutants in vivo (Fig. 7C-E). These results indicate a potential therapeutic window to combine clinically relevant doses of Menin- and immunoproteasome-inhibitors to increase therapeutic efficacy and potentially prevent outgrowth of Menin-inhibitor resistant clones.

## Discussion

Leukemic cells show aberrant regulation of the epigenetic machinery to activate and maintain transcriptional programs required for cell competition, proliferation and self-renewal. Leukemias that harbor balanced translocations involving the KMT2A-gene locus show a unique disease biology and particularly poor prognosis [1, 32] due to molecular persistence and a high relapse rate. Direct targeting of the oncogenic fusion has not been successful so far [1]. Most recently, inhibitors of the KMT2A-complex members Dot1L and Menin have shown promising activity in pre-clinical studies [3, 5, 6]. However, clinical use of the chromatin complex inhibitors has been hampered by their bioavailability, limited efficacy [10] and emergence of resistance mediating mutations [33].

As the presence of the KMT2A-fusion may create secondary dependencies, we aimed to explore the proteomic landscape of KMT2A-r leukemia. Proteostasis appeared as an important cellular function enriched in KMT2A::MLLT3 driven murine AML. Moreover, catalytic proteasome subunits were highly expressed in human KMT2A-r AML, a finding that had been reported by others before [17] without providing functional or mechanistic explanation. Here, we provide first evidence, that inactivation of catalytic immunoproteasome subunit PSMB8 results in impaired cellular proliferation of KMT2A-r leukemia.

**Fig. 8:**
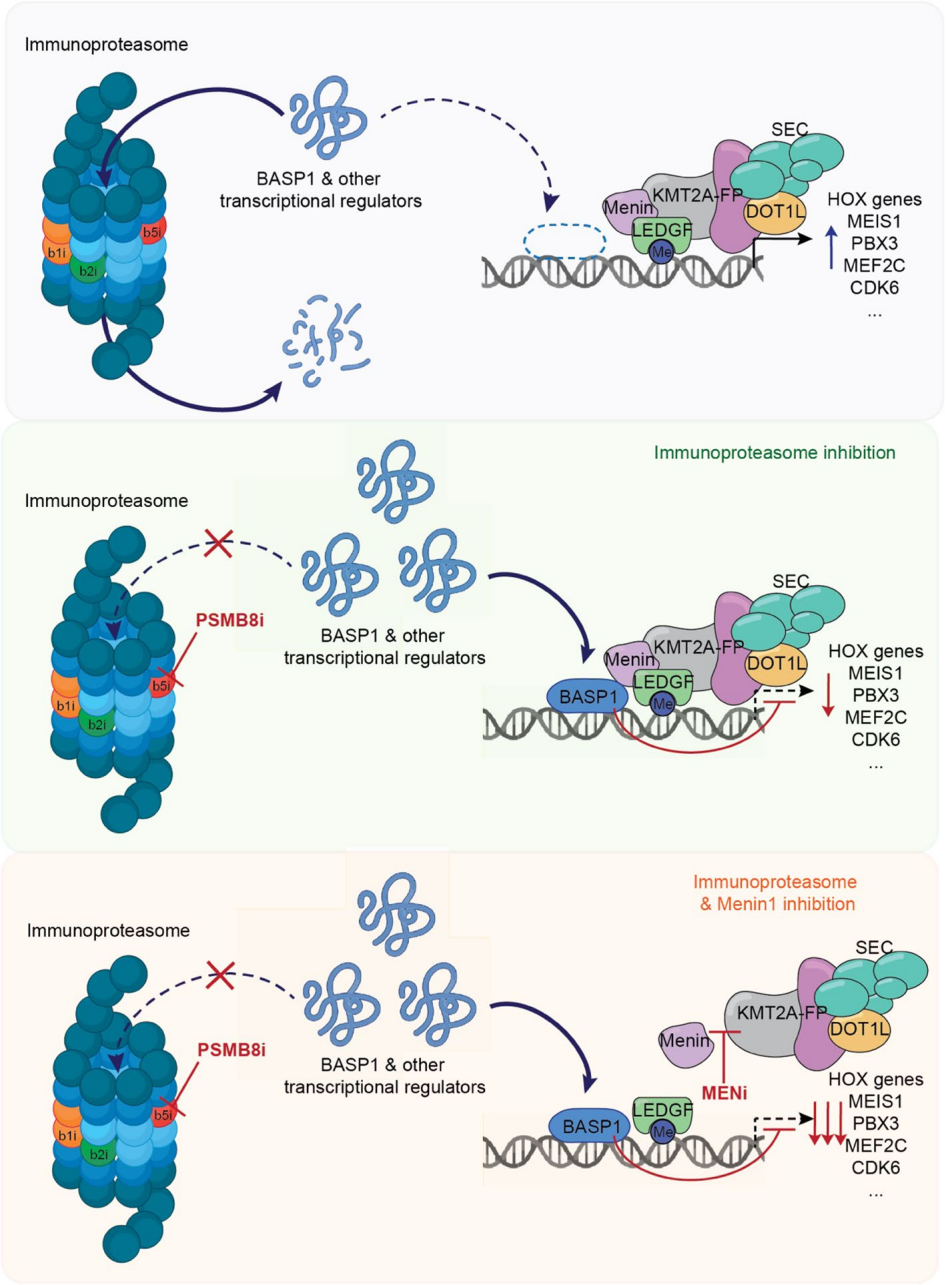
Schematic depicting the relevant mechanisms of PSMB8 inhibition as a functional vulnerability in KMT2A-r leukemia through repressive nuclear functions of BASP1 in conjunction with Menin-inhibitor treatment

The immunoproteasome is a specialized version of the proteasomal catalytic core particle [12, 34], where catalytic subunits of the standard proteasome can be rapidly substituted by their immunoproteasome counterparts PSMB8 (LMP7, beta5i), PSMB9 (LMP2, beta1i) and PSMB10 (MECL-1, beta2i) to increase specificity and efficacy in a context dependent manner. Use of the covalent and specific PSMB8-inhibitor PR-957 (ONX-0914) prevented disease progression in models of autoimmune disorders, without evidence of significant toxicity [20, 35]. Immunoproteasome inhibitors have demonstrated their efficacy and safety against inflammatory and autoimmune diseases, even though their development for the treatment of hematologic malignancies is still in the early phases. Recently, M3258 was synthesized using the α-aminoboronic acid scaffold as a starting point to optimize selectivity for PSMB8 (> 500fold over PSMB5) and has entered early clinical trials for multiple myeloma [14, 36, 37]. In contrast, approved compounds such as bortezomib or carfilzomib inhibit standard- and immunoproteasome subunits in a rather non-specific manner leading to increased hematologic toxicity [14]. While effects of immunoproteasome inactivation on antigen presentation and T-cell activation have been investigated in very detail (reviewed in [12]), our data describes a so far unidentified cell-intrinsic mechanism of immunoproteasome inhibition in the context of KMT2A-r AML: pharmacologic inactivation of its catalytic subunit PSMB8 results in increased abundance of the transcription factor BASP1 and leads to repression of KMT2A-target genes (Fig. 8). BASP1 has been described as a repressive transcriptional co-factor in conjunction with several transcriptional regulators such as WT, Prohibitin, MYC or ERalpha [38–40] that may modulate chromatin accessibility [41] and also exerts cytoplasmatic functions [42, 43]. Depending on the cellular context, both, oncogenic and tumor suppressor functions of BASP1 have been described. Exploiting this alternative mechanism of targeting transcriptional oncogenic networks, we demonstrate that combination of pharmacologic PSMB8- and Menin-inhibition results in synergistic abrogation of human leukemic cells in vivo and improved elimination of leukemia initiating cells in pre-clinical PDX-models when compared to Menin-inhibitor treatment alone. The enhanced effect by inhibiting both molecules is preserved across the class of (immuno-) proteasome inhibitors irrespective of their specificity and for second-generation clinical grade Menin-inhibitors. Although both types of inhibitors reduce proliferative capacity and self-renewal capacity of leukemic stem cells (rather than induction of apoptosis), combination of both classes may facilitate eradication of residual leukemic clones through mechanisms of cell competition and induction of differentiation.

While identified as a selective functional vulnerability in KMT2A-r cells, inactivation of PSMB8 attenuated growth of NPM1-mutated cells, suggesting activity in this frequent subtype of AML. This finding is consistent with recent findings that mutated NPM1 binds to KMT2A co-occupied targets, which explains its sensitivity to Menin-inhibitor treatment [44]. Most recently, a phase 1 trial investigating the Menin-inhibitor Revumenib (SNDX-5613) in KMT2A-r and NPM1c AML reported on emergence of MEN1 mutations mediating resistance in 38.7% of patients [33]. While this report emphasizes the evolution of resistance mutants against a chromatin-targeting therapy, the non-epigenetic mechanism of inhibiting oncogenic gene expression through repression of KMT2A-target genes described here is still effective in KMT2A-r, MEN1-mut cells.

## Conclusion

Thus, combined use of immunoproteasome- and Menin-inhibitors facilitates efficient drug targeting of oncogenic transcriptional networks in AML (Fig. 8) and provides the opportunity for improved reduction of disease burden and preserved activity against MEN1-mutated clones to prevent resistance to targeted epigenetic therapies.

## Material & methods

### Cell lines

Human AML cell lines were purchased from DSMZ (Braunschweig, Germany). Cas9-Blast or deadCas9-Blast (dCas9) cell lines are subclones of the respective cell lines and stably transduced with lentiCas9-Blast (Addgene #52962) or dCas9-VP64-Blast (Addgene #61425) and MS2-P65-HSF1-Hygro (Addgene #61426). MV-4;11 M327I and MV-4;11 T349M have been previously described [33]. Cells were cultured in RPMI 1640 medium (Thermo Fisher Scientific, Waltham, USA) supplemented with 10-20% FBS (Thermo Fisher Scientific) in 5% CO2 at 37°C.

### Animal studies

All mice were housed under pathogen-free conditions in the Animal Research Facility of the Otto-von-Guericke University Medical Faculty Magdeburg, the ZET (University Hospital Jena) and ZTL (University Medicine Greifswald). All experiments were conducted after approval by the respective authorities of Sachsen-Anhalt (42502-2-1279 UniMD), Thüringen (02-030/2016) and Mecklenburg-Vorpommern (7221.3-1-019/22). Conventional LMP7 -/- and i-KMT2A::MLLT1 mouse models have been previously published [24, 45]. For induction of KMT2A::MLLT1 expression in the i- KMT2A::MLLT1 model, mice were treated for 2 weeks with food supplemented with Doxycycline (DOX; 0.545 g/kg). For induction of KMT2A::MLLT3 driven leukemia we used established retroviral MSCV-GFP-based vectors to express KMT2A::MLLT3 in hematopoietic stem- and progenitor cells (Lineage^-^Sca1^+^c-Kit^+^, LSK cells) as described before. For competitive repopulation assays of normal hematopoiesis, 2x10^6^ BM cells of 6-8-weeks old B6.SJL-PtprcaPepcb/BoyCrl (CD45.1) or C57BL/6JRj (CD45.2) animals (Janvier Labs) and 2x10^6^ (CD45.1/2) competitor cells (derived from intercrossing B6.SJL-PtprcaPepcb/BoyCrl (Charles River) with C57BL/6JRj (CD45.2) animals) were transplanted via lateral tail vein injection into lethally irradiated (13 Gy, single-dose) 6-8-weeks old C57BL/6JRj (CD45.2) recipient mice or B6.SJL-PtprcaPepcb/BoyCrl (CD45.1) (females), respectively.

### Primary patient samples

Primary AML patient samples and healthy donor controls were obtained after informed consent and according to the Helsinki declaration within the AML-trials of the German-Austrian AMLSG study group and from the Hematology Tumor Bank Jena and Magdeburg, approved by the respective local ethics committee (Ethics Committee of the Medical Faculty, University Hospital Jena #4753/04-16 or University Hospital Magdeburg #115/08). Human bone marrow aspirates were separated with Ficoll-Paque Plus (GE Healthcare, Chicago, IL, USA) following the manufacturer`s instruction and cryopreserved in 1x freezing medium (80% FBS + 10% DMSO + 10% IMDM medium).

### Mouse xenotransplantation

NOD.Cg-Prkdc^scid^ Il2rg^tm1Wjl^ Tg(CMV-IL3, CSF2, KITLG) 1Eav/MloySzJ (NSGS) mice were obtained from The Jackson Laboratory (Bar Harbor, ME, USA). NOD.Cg-Prkdc^scid^ Il2rg^tm1Wjl^/RJ (NXG) mice were obtained from Janvier (Le Genest-Saint-Isle, France). 8-12-weeks old adult mice (male and female) were irradiated with 2 Gy (single dose) before transplantation. 1x10^5^ MOLM-13, ML-2 or MV-4;11 cells transduced with either of two PSMB8 shRNAs or non-targeting control; with a pLEX vector containing the sequence of BASP1 or an empty vector; or treated *ex vivo* with the indicated inhibitors were injected intravenously via the tail vein. For patient derived xenograft experiments (PDX) of human KMT2Ar leukemia, 1-5x10^4^ cells from patient samples containing an KMT2A::MLLT3or KMT2A::AFF1 translocation were injected into NOD.Cg-^KitW-41J^Prkdc^scid^Il2rg^tm1Wjl^/WaskJ (NSGW41) [46]. Human myeloid engraftment (hCD45+) was analyzed by flow cytometry.

### In vivo drug treatment

PR-957 (MedChemExpress, Monmouth Junction, NJ, USA) was solved in DMSO and diluted in CAPTISOL^®^ from a sterile stock solution and administered by i.v. injections (3mg/kg, 6mg/kg or 10mg/kg as indicated) once daily as published before [20]. NaCl0.9% was injected as diluent control. MI-503 (Selleckchem, Houston, TX, USA) was dissolved in 25%DMSO/25%PEG-400/50%NaCl0.9% or a diluted solution of DMSO in CAPTISOL^®^and administered i.p. at 50mg/kg versus NaCl0.9% or 30%DMSO-70%NaCl0.9% as diluent control.

### Methylcellulose colony-forming assays

Primary mouse bone marrow (BM) cells (transduced with MA9/KRAS, MA6 or AML1-ETO/KRAS) were seeded in MethoCult GF M3434 (Stemcell Technologies, Vancouver, Canada) at a concentration of 1x10^3^ cells/replicate. LSK cells isolated from LMP7 ^-/-^ or LMP7 ^+/+^mice (transduced with KMT2A::MLLT3 or KMT2A::MLLT1) were seeded at 500 cells/replicate. Colonies propagated in culture were scored on day 7 and replated for 4 weeks. For re-plating of BM cells, colonies were harvested from the methylcellulose medium, washed with PBS/1%FBS and re-seeded at the same concentration. Human AML cell lines infected with either NT or PSMB8-specific shRNAs were plated at 250 cells/replicate in MethoCult H4230 (Stemcell Technologies) supplemented with 10% FBS. Colony numbers were counted on day 10-14.

### Immunohistochemistry

Spleen, liver and lung were fixed in 4% paraformaldehyde for 24 hours followed by incubation in 30% ethanol for 30 min and 50% ethanol for 24 hours. Organs were embedded in paraffin and paraffin sections were cut on a rotary microtome (Microm HM 355S, Thermo Fisher Scientific), mounted on microscope slides and air-dried in an oven at 37°C overnight. Tissue section slides were then processed automatically for H&E staining (Leica AutoStainer XL, Leica Biosystems, Wetzlar, Germany). Images were acquired at 10x magnification on an AxioImager A.2 (Carl Zeiss Microscopy, Jena, Germany). Images were processed using the ImageJ software (NIH, Bethesda, MD, USA).

### Flow cytometry and antibody staining

Immunophenotyping of immature and mature cell compartments and of leukemic cells was performed as described before [19]. The antibodies used for cell surface staining are provided in Table S1. Cells were stained in PBS/1% FBS for 1.5 hours at 4°C. SYTOX^®^Blue Dead Cell Stain (Life Technologies, Darmstadt, Germany) was used to exclude dead cells. Flow cytometry was performed on a LSRFortessa or FACS Canto II cytometer (BD Biosciences, Franklin Lakes, NJ, USA). Cell cycle analysis was performed using the Click-iT™ EdU Alexa Fluor™ 647 Assay Kit (Thermo Fisher Scientific) as per the manufacturer`s instructions.

### Genetic inactivation by RNAi

The pLKO.1-vector system with puromycin resistance gene was used. HEK293T cells were transfected using FUGENE^®^HD Transfection Reagent (Promega, Madison, WI, USA) to generate lentiviral particles as described before [19]. Target cells were infected twice (8 hours gap) by spinfection (872g, 1.5 h, 33°C). Puromycin selection (1 µg/ml) was started at 48h. shRNA sequences are provided in Table S2.

### CRISPR activation

Guide RNAs were designed using the CRISPick tool (Broad Institute, https://portals.broadinstitute.org/gppx/crispick/public). sgRNA sequences: Luciferase_sgRNA (GATTCTAAAACGGAT-TACCA), sgRNA3a BASP1 (CGGGGAGCGCGGGAGGAGGG), sgRNA5a BASP1 (GGGCGGGGAGCGCGG-GAGGA). For cloning sgRNA sequences, the improved-scaffold-pU6-sgRNA-EF1Alpha-PURO-T2A-RFP (ipUSEPR) vector system was used. HEK293T cells were transfected using FUGENE^®^HD Transfection Reagent (Promega, Madison, WI, USA) to generate lentiviral particles as described before [19]. Cell lines stably expressing Cas9 were infected twice (8 hours gap in between) by spinfection (872xg, 1.5 hours, 33°C). The cells expressing sgRNAs were selected with 1 µg/ml puromycin starting on day 2 post-infection. Cells were collected for RT-qPCR on day 8 post-infection

### Cellular proliferation & apoptosis

Cellular proliferation was assessed by cell counting using Trypan Blue exclusion. Apoptosis was measured by flow cytometry using Annexin V/ SYTOX^®^Blue staining.

### Genome-wide CRISPR/Cas9 screening

6x10^8^ MOLM-13 cells were transfected (872 g, 37C, 2h) with lentiviral particles containing the human lentiviral CRISPR/Cas9 library (developed and kindly provided by Dr. X. S. Liu (Addgene, #1000000132)). Cells were treated for 12 days with increasing concentrations of PR-957 (50-200nM) or DMSO as a control. Genomic DNA was extracted, and library amplification performed according to standard protocols (https://www. addgene.org/pooled-library/liu-crispr-knockout/).

### Quantitative real-time PCR

1µg of total RNA were extracted using TRIzol Reagent (Thermo Fisher Scientific,) or the RNeasy Mini Kit (Qiagen, Hilden, Germany). RNA was reverse-transcribed using Omniscript RT Kit (Qiagen, Hilden, Germany) as per the manufacturer`s instructions and complementary DNA samples were analyzed by RT-qPCR using SYBR^®^ Premix Ex TaqII™ (Clontech Laboratories, Mountain View, CA, USA) following the manufacturer’s instruction. Gene-specific primers were designed to span exon-exon boundaries. All expression values were normalized and standardized using the Bio-Rad CFX Manager software (Munich, Germany) and presented as fold changes of gene expression in the test sample compared to the control. Primer sequences are listed in Table S4.

### Protein extraction and immunoblotting

Cells were washed twice with ice-cold PBS and lysed in lysis buffer (50 mM HEPES pH7.4, Glycerol 10%, NaCl 150 mM, TritonX-100 1%, MgCl 1.5 mM, EGTA 5mM) for 1 hour on ice. For nuclear extraction, NE-PER™ Nuclear and Cytoplasmic Extraction Kit (Thermo Fisher Scientific) was used following the manufacturer´s instructions. Protein concentration was calculated using the Protein Assay Dye Reagent Concentrate (Bio-Rad Laboratories, Inc., Hercules, CA, USA) following the manufacturer’s instruction. HRP-conjugated anti-rabbit or anti-mouse secondary antibodies were purchased from Cell Signaling (Denvers, MA, USA). Primary antibodies used included: anti-BASP1 (Thermo Fisher Scientific, 703692), anti-c-Myc (Abcam, Cambridge, UK; ab32072), anti-FLT3 (Cell Signaling, CS3462), anti-GAPDH (Meridian Life Sciences, Cincinnati, OH, USA; H68504M), anti-HA-Tag (Cell Signaling, CS3724), anti-HDAC1 (Cell Signaling, CS5356), anti-MEF2C (Cell Signaling, CS5030S), anti-PBX3 (Abcam, ab109173), anti-PSMB5 (Santa Cruz Biotechnology, Dallas, TX, USA; sc393931), anti-PSMB8/LMP7 (Abcam, ab3329 / Santa Cruz Biotechnology, sc365699), anti-PSMB9/LMP2 [47], anti-PSMB10/MECL1 (Thermo Fisher Scientific, PA5-19146), anti-Vinculin (Sigma Aldrich, St Louis, MO, USA; V9131), anti-β-actin (Santa Cruz Biotechnology, sc47778).

### Global proteome analysis

For global proteome profiling, leukemia development was initiated with KMT2A::MLLT3 (or AML1-ETO as control) containing MSCV-GFP constructs. Murine stem-and progenitor cells (LSK cells: Lin^-^Sca^+^c-Kit^+^) from 6-8 weeks-old C57BL/6J donors (females) were sorted and infected by co-localization of virus supernatant (containing one of the oncogenes) on retronectin-coated plates. 72 hours after infection equal numbers of GFP+ cells were injected into sublethally irradiated recipient hosts (7Gy). 2x 10^5^ LSC-enriched (GFP^+^ c-Kit^+^) cells (4 replicates per oncogene) were sorted directly into 2x lysis buffer (for a final concentration: 1% SDS, 50 mM HEPES, pH 8.5, 10 mM DTT; volume of lysis buffer added to collection tube was estimated to be equal to the volume of the sheath buffer). For global proteome profiling of human AML cell lines, 2x10^6^ cells treated with 100nM PR-957 or DMSO, 72h (4 replicates per treatment). Sample processing was performed as described previously [48].

### ATAC-sequencing

5x10^4^ MOLM-13 cells were used for nuclear extraction. Nuclear fractions were tagmented using Illumina Tagment DNA TDE1 Enzyme and Buffer Kit (New England Biolabs® (NEB), Ipswich, MA, USA). Subsequently, DNA was extracted using the DNA Clean & Concentrator-5 (Zymo Research, Irvine, CA, USA). DNA was mixed with a universal i5 and uniquely barcoded i7 primer and amplified using NEB-Ultra II Q5 master mix (New England Biolabs, M0544S) in a thermocycler using the following conditions: 98°C for 30 seconds; 7 cycles of 98°C for 10 seconds, 63°C for 30 seconds; and 72°C for 1 minute. Post amplification libraries were size selected at >250bp in length using SPRI-select beads (Beckman Coulter, Brea, CA, USA). Library fragment length was checked by HSD5000 Tape (Agilent, Santa Clara, CA, USA) and DNA concentration was determined by the Qubit dsDNA HS Assay Kit (Thermo Fisher Scientific).

### Cut & Tag-sequencing

Protein A fused to Tn5-transposase was expressed and purified using a publicly available construct (Addgene #124601). In brief, 1x10^5^ MOLM-13 cells were washed in Wash Buffer (20 mmol/L HEPES pH 7.5, 150 mmol/L NaCl, 0.5 mmol/L Spermidine, protease inhibitor cocktail) and bound to Concanavalin A beads (Bangs Laboratories, Fishers, NC, USA; BP531). Cells were resuspended in 50μL Digitonin Wash Buffer (20 mmol/L HEPES pH 7.5, 150 mmol/L NaCl, 0.5 mmol/L Spermidine, protease inhibitor cocktail, 2 mmol/L EDTA, 0.05% Digitonin) and incubated with anti-H3K4me3, -H3K4me1, -H3K27ac, -and H3K27me3 antibody at a 1:100 dilution overnight at 4°C. Beads were resuspended in 100μL Digitonin Wash Buffer with a secondary antibody diluted 1:100 and incubated for 30min at room temperature.  Cells were washed three times in Digitonin Wash Buffer and resuspended in Digitonin-300 Buffer (0.05% Digitonin, 20 mmol/L HEPES, pH 7.5, 300 mmol/L NaCl, 0.5 mmol/L Spermidine, protease inhibitor cocktail) containing 1:250 pA-Tn5 transposase and incubated at room temperature for 1 hour. Subsequently, cells were washed three times in Digitonin-300 Buffer and resuspended in 100 μL Tagmentation Buffer (10 mmol/L MgCl2 in Digitonin-300 Buffer) and incubated at 37°C for 1 hour. Tagmentation was stopped by adding 10μL of 0.5 M EDTA, 3μL of 10% SDS, and 2.5 μL of 20 mg/mL Proteinase K (Thermo Fisher Scientific, 25530049), and samples were incubated 37°C overnight. Tagmented DNA was purified using AMPureXP-beads (Beckman Coulter). For each sample, 21μL DNA was mixed with a universal i5 and uniquely barcoded i7 primer and amplified using NEBNext High Fidelity 2x PCR Master Mix (New England Biolabs, M0541S) in a thermocycler using the following conditions: 98°C for 30 seconds; 14 cycles of 98°C for 10 seconds, 63°C for 10 seconds; and 72°C for 2 minutes. DNA was purified with AMPureXP beads and quality was assessed by the Qubit dsDNA HS Assay Kit and HSD5000 Tape.

### Cut & Run-sequencing

6x10^5^ MOLM-13 cells were harvested per reaction. Preparation of the samples was performed using CUTANA^TM^ ChIC/CUT&RUN Kit (EpiCypher, Chapel Hill, NC, USA) following the manufacturer´s instructions. Antibodies used: anti-BASP1 (Thermo Fisher Scientific, 703692), IgG Control (EpiCypher, 13-0042k) and H3K4me3 (positive control) (EpiCypher, 13-0041k). Library amplification was done using the NEBNext® Ultra™ II DNA Library Prep Kit for Illumina® (NEB) using 6ng of sample. Post amplification libraries were size selected at 150-250bp in length using AMPure beads (Beckman Coulter). Library fragment length was checked by HSD1000 Tape and DNA concentration was determined by Qubit.

### Statistics and analysis of sequencing and proteome data

Statistical analyses were performed using Student’s *t*test or 2-way ANOVA (normal distribution) or Mann-Whitney U test (when normal distribution was not given). *p*less than 0.05 was considered statistically significant. mRNA expression data of the catalytic (immuno-) proteasome subunits in different genetic AML subtypes was downloaded from BloodSpot data base [49] (http://servers. binf.ku.dk/bloodspot/) and protein expression data from depmap.org. Detailed information on the analyses of proteome, RNA-, ATAC-, Cut&Tag-, Cut&Run-sequencing data is provided in the Supplementary Material and Methods.

### Supplementary Information

**Additional file 1: Fig. S1.** (A) Western Blotting showing expression of PSMB8, PSMB9 and PSMB10 in KMT2A-r (ML-2, THP-1, MV-4;11, KOPN-8, MOLM-13) and non-KMT2A-r (HL-60, K-562) cell lines. Hela cells were used as a negative control, since they do not express immunoproteasome, and T cells as a positive control. (B) Violin plots displaying log2-fold protein expression as assessed by proteome analysis (Jayavelu et al., Cancer Cell, 2022; PXD022894) of PSMB8, PSMB9 and PSMB10 in different KMT2A-r and non-KMT2A-r cell lines. Mann-Whitney U test. (C) CRISPR-Cas9 cell competition assay showing the effect of deletion of each catalytic immunoproteasome subunit over time. Catalytic immunoproteasome subunits were knocked out using different single guide RNAs (5 specific for PSMB8; 3 specific for PSMB9 and PSMB10, respectively) in human KMT2A-r AML cells (MOLM-13) and the chimerism of knockout and wildtype cells over time is visualized in the graphs. Each blue line represents the chimerism at day 0, 3, 6, 9, and 12. A decrease in the % of RFP+ cells (shown on the Y axis) over time, as seen most pronounced for PSMB8, reflects a competitive disadvantage of cells that harbor the respective knockout. Single guide RNAs (red lines) against the essential gene RPA3 were used as a positive control and non-targeting guide against Luciferase as a negative control (black line). (D) Percentage of Annexin^+^ cells (containing Annexin^+^-SYTOX^®^Blue^+^ and Annexin^+^-SYTOX^®^Blue^-^ populations measured by flow cytometry) in KMT2Ar cell lines (MOLM-13, THP-1, MONO-MAC-6, KOPN-8, ML-2) transduced with shRNAs targeting PSMB8 or a non-targeting control (shNT) at day 6 post-infection. *n*=3-5 independent experiments; mean with SD; paired Student t test. (E) Growth curves depicting cell counting after trypan blue exclusion of MOLM-13 cells and MOLM-13 PSMB8 overexpressing cells (+PSMB8) transduced with shRNAs targeting PSMB8 or a non-targeting control (shNT). *n*=3 independent experiments; mean with SD. (F) Representative pictures of colonies from MOLM-13 and ML-2 cells transduced with PSMB8 shRNAs or shNT. Scale bars, 200 µm. Representative Western Blotting plots of MOLM-13 and ML-2 cells confirming PSMB8 deletion at day 4 post-infection before transplantation into NSGS recipient mice. (G) Representative Western Blotting plots of MOLM-13 and ML-2 cells confirming PSMB8 deletion at day 4 post-infection before transplantation into NSGS recipient mice. **Fig. S2.** (A) Violin plots showing the percentage of cells detected in S phase in MOLM-13, ML-2, KOPN-8 and MV-4;11, cells after treatment with 100nM PR-957 or DMSO for 24 hours. Samples were labeled for flow cytometry-based analysis of cell cycle using the Click-iT® EdU assay. *n*=3 independent experiments; paired Student t test. (B) Representative flow cytometry plots from Click-iT® EdU assay. (C) Schematic representation of patient derived xenografts (PDX). (D) Violin plots depicting % of hCD45+ cells in peripheral blood in PDX models of KMT2A::MLLT3 and KMT2A::AFF1. 2-way ANOVA. (E) Violin plots of hCD45+ cells in spleen and bone marrow at the time of sacrifice. Mann-Whitney U test. **Fig. S3.** (A) Serial re-plating to assess colony formation in methylcellulose using murine LSK (Lin^-^ Sca1^+^ c-Kit^+^) cells isolated from LMP7^+/+^ or LMP7^-/-^ mice and transformed with KMT2A::MLLT3 or KMT2A::MLLT1. *n*=4 independent experiments; mean with SD; paired Student t test. (B) Schematic representation showing the transplantation of KMT2A::MLLT3 retrovirally transformed LSKs from LMP7^+/+^ or LMP7^-/-^ mice. (C) Schematic representation describing the transplantation of i-KMT2A::MLLT1 BM cells lentivirally transduced with shRNA1 or shRNA4 against LMP7 or a non-targeting control (shNT) into CD45.1 recipient mice. Diet of the donor mice was supplemented with Doxycycline (DOX; 0,545 g/kg) for 2 weeks to induce KMT2A::MLLT1 expression. Recipient mice were also kept with DOX supplemented food. (D) Violin plots depicting percentage of CD45.2+ cells in peripheral blood of CD45.1 recipient mice from sh1 LMP7 (*n*=4), sh4 LMP7 (*n*=4) or shNT (*n*=4) transformed i-KMT2A::MLLT1. One cohort; 2-way ANOVA. (E) Kaplan-Meier survival curves of CD45.1 recipient mice transplanted with 1x10^6^ cells as shown in S3C. One cohort; Mantel-Cox test. (F) Spleen Colony Formation Assay in vivo (CFU-S12): Spleen colony numbers 12 days after injection of 100 LSK cells isolated from LMP7^+/+^ (*n*=10) or LMP7^-/-^ (*n*=10) mice. Two independent cohorts; Mann-Whitney U test (G) Schematic representation depicting competitive repopulation assay to investigate the effects of LMP7 depletion on normal hematopoietic stem- and progenitor cells (HSPCs). **Fig. S4.** (A) Representative pictures of colonies from murine LSK cells transformed with KMT2A::MLLT3/KRAS, KMT2A::MLLT4 or AML1-ETO/KRAS at week 2. Scale bars, 200 µm. (B) Schematic representation of *in vivo* PR-957 vs NaCl 0.9% treatment in C57BL/6 mice transplanted with KMT2A::MLLT3 leukemic cells. Subsequent secondary transplantation of whole bone marrow cells into secondary recipients. (C) Pictures of tissue sections from liver, lung and spleen of *in vivo* PR-957 vs NaCl treated mice at the time of sacrifice. (D) Representative flow cytometry plots with the gating strategy for the analysis of granulocyte-macrophage progenitors (GMPs), megakaryocyte-erythroid progenitors (MEPs), common myeloid progenitors (CMPs), hematopoietic stem cells (HSCs) and multipotent progenitors (MPPs) in the competitive repopulation assay. FSA: Forward Scatter Area; SSA: Side Scatter Area; FSH: Forward Scatter Height; FSW: Forward Scatter Width; SSH: Side Scatter Height; SSW: Side Scatter Width; LK: Lineage- cKit+; LSK: Lineage- Sca1+ cKit+. **Fig. S5.** (A) Volcano plot of differentially regulated genes in RNA-sequencing. 100nM PR-957 vs. DMSO, 72h, MOLM-13. Upregulated (red; FC>1.5, *p*<0.05) and downregulated (blue; FC<-1.5, *p*<0.05). (B) Heatmaps displaying H3K27ac, H3K27me3, H3K4me1 and H3K4me3 Cut&Tag signal mapping to a 2-kb window around TSS. 100nM PR-957 vs. DMSO, 48h, MOLM-13. (C) Integrative Genomics Viewer (IGV) tracks from Cut&Tag-sequencing data in MOLM-13 cells depicting binding of H3K27ac, H3K27me3, H3K4me1 and H3K4me3 after DMSO or PR-957 treatment at HOXA and MEIS1 loci. (D) Volcano plot of differentially accessible regions in ATAC-seq. 100nM PR-957 vs. DMSO, 72h, MOLM-13. Upregulated (red; FC>2, *p*<0.05) and downregulated (blue; FC<-2, *p*<0.05). (E) Stacked bar plot depicting genomic distribution of PSMB8 ATAC-seq peaks. 100nM PR-957 vs. DMSO, 72h, MOLM-13. (F) Schematic representation of the genome-wide CRISPR-Cas9 screen. (G) Volcano plots showing the distribution of genes being enriched (positive beta-scores) or depleted (negative beta-scores) in the genome-wide CRISPR-Cas9 screen in PR-957 treated MOLM-13 cells and DMSO diluent control. (H-I) Western Blotting showing expression of BASP1 in nuclear (N) and cytoplasmic (C) fractions of MOLM-13, MV-4;11, KOPN-8 and ML-2 cells. (H) BTZ (4nM) vs. DMSO, 72h. (I) CFZ (4nM) vs. DMSO, 72h. (J) Integrative Genomics Viewer (IGV) tracks from Cut&Run-sequencing data in MOLM-13 cells depicting binding of BASP1 after DMSO or PR-957 treatment and of H3K4me3 after DMSO treatment at the KMT2A, MYC, JUN and GNAS loci. **Fig.** **S6.** (A) Western Blotting confirming BASP1 overexpression after transduction with a pLEX vector containing the sequence of human BASP1 with an HA tag (BASP1) or an empty vector with the HA tag (EV) as a control in MOLM-13, MV-4;11, ML-2 and KOPN-8 cells. Samples were collected at day 6 post-infection. (B) Percentage of Annexin^+^ cells of KMT2A-r cell lines overexpressing BASP1 or control cells at day 6 post-infection. *n*=4 independent experiments; mean with SD; paired Student t test. (C) Growth curves of MOLM-13-deadCas9 (MOLM-13-dCas9) and MV-4;11-dCas9 cells containing the MS2-P65-HSF1 activator helper complex transduced with sgRNAs designed to target the promoter region of BASP1 (sgRNA3a BASP1 and sgRNA5a BASP1) or sgRNA Luciferase as a negative control. *n*=4 independent experiments; mean with SD; 2-way ANOVA. (D) Relative BASP1 mRNA expression in BASP1-overexpressing cells (MOLM-13-dCas9 and MV-4;11-dCas9) compared to sgRNA Luciferase cells assessed by Real Time Quantitative PCR (RT-qPCR). *n*=4 independent experiments; mean with SD. (E) Western Blotting confirming BASP1 overexpression after transduction with pLEX-BASP1 (BASP1) or pLEX-EV (EV) in MOLM-13 and MV-4;11 cells. Samples were collected 96 hours after transduction and before transplantation into recipient mice. **Fig. S7.** (A) Western Blotting quantification of c-MYC, MEF2C, FLT3 (mature), FLT3 (premature), PBX3, PSMB5 and PSMB8 protein expression in MOLM-13 cells treated with 100nM PR-957, 1μM MI-503, a combination of both or DMSO. *n*=3-4 independent experiments; mean with SD; paired Student t test, (B) Percentage of Annexin^+^ cells in MOLM-13, MV-4;11, ML-2, KOPN-8 and OCI-AML3 cells after treatment with 20nM PR-957, 200nM MI-503, a combination of both or DMSO as diluent control at day 8 post-infection. *n*=5 independent experiments; mean with SD; paired Student t test. (C) Kaplan-Meier survival curves of NXG recipient mice transplanted with limiting numbers (2x10^6^, 2x10^5^, 2x10^4^) of whole bone marrow cells from *in vivo* treated mice (DMSO, MI-503, MI-503 + PR-957). Two independent cohorts; Mantel-Cox test. (D) Violin plots showing percentage of hCD45^+^ cells in bone marrow (BM) at the time of sacrifice. **Fig. S8.** (A) Negative selection cell competition assay using MV-4;11 Menin-wildtype (BFP^+^) cells and resistant MV4;11 Menin M327I or T349M (RFP^+^) cells (as published in Perner et al. Nature 2023). Chimerism of RFP^+^ mutant clones over BFP^+^ Menin-wildtype cells is visualized for a total of 9 days upon exposure to PR-957(100nM), Revumenib (50nM), MI503 (1μM) or DMSO as a control.** Table S1.** Flow cytometry and western blot antibodies used in this study.** Table S2.** shRNA sequences used in this study. **Table S3.** sgRNAs for CRISPRa used in this study. **Table S4.** Primer sequences for RT-qPCR used in this study.

## Data Availability

Raw data files for the RNA-sequencing, ATAC-sequencing, Cut&Run and Cut&Tag analysis have been deposited in the NCBI Gene Expression Omnibus (GEO) under accession number GSE225391. The mass spectrometry data have been deposited to the ProteomeXchange Consortium (http://proteomecentral.proteomexchange.org) via the PRIDE partner repository with the dataset identifier PXD041245.

## Abbreviations

AE: AML1-ETO9a
ALL: Acute Lymphoblastic Leukemia
AML: Acute myeloid leukemia
BASP1: Brain Abundant Membrane Attached Signal Protein 1
BM: Bone marrow
BTZ: Bortezomib
C: Cytoplasmic
CFZ: Carfilzomib
CI: Confidence intervals
CMP: Common myeloid progenitors
DOX: Doxycycline
ELDA: Extreme Limiting Dilution Assay
FC: Fold change
GMP: Granulocyte–macrophage progenitors
GSEA: Gene set enrichment analysis
HGB: Hemoglobin
hCD45^+^: Human CD45^+^
HSC: Hematopoietic stem cell
HSPCs: Hematopoietic stem- and progenitor cell
IP: Immunoproteasome
KMT2A-r: KMT2A-rearranged
LD: Limiting dilution
LSC: Leukemic stem cell
LSK: Lineage^−^ Sca1^+^ c-Kit^+^
LT-HSC: Long-term HSC
MA9: MLL-AF9
MEN1: Menin
MEP: Megakaryocyte-erythroid progenitors
MLL: Mixed-Lineage-Leukemia
MPP: Multipotent progenitor
MS: Mass spectrometry
N: Nuclear
NES: Normalized enrichment score
NPM1c: NPM1-mutant
NSGW41: NOD.Cg^KitW^^−^^41J^Prkdc^scid^Il2rg^tm1Wjl^
NXG: NOD.Cg-Prkdc^scid^ Il2rg^tm1Wjl^/RJ
PB: Peripheral blood
PDX: Patient derived xenografts
PLT: Platelets
Prog: Progenitor cell abundance
SD: Standard Deviation
SEM: Standard Error of Mean
shNT: Non-targeting control
ST-HSC: Short-term HSC
TSS: Transcription Start Site
UPS: Ubiquitin–proteasome system
WBC: White blood counts
WT: Wild-type
