# Supplementary material for: Immunoproteasome function maintains oncogenic gene expression in KMT2A-complex driven leukemia
Source: Mol Cancer. 2023 Dec 4;22:196. doi: 10.1186/s12943-023-01907-7 (PMC10694946; doi:10.1186/s12943-023-01907-7)
Supplement: Supplementary file 1 — Additional file 1: Fig. S1. (A) Western Blotting showing expression of PSMB8, PSMB9 and PSMB10 in KMT2A-r (ML-2, THP-1, MV-4;11, KOPN-8, MOLM-13) and non-KMT2A-r (HL-60, K-562) cell lines. Hela cells were used as a negative control, since they do not express immunoproteasome, and T cells as a positive control. (B) Violin plots displaying log2-fold protein expression as assessed by proteome analysis (Jayavelu et al., Cancer Cell, 2022; PXD022894) of PSMB8, PSMB9 and PSMB10 in different KMT2A-r and non-KMT2A-r cell lines. Mann-Whitney U test. (C) CRISPR-Cas9 cell competition assay showing the effect of deletion of each catalytic immunoproteasome subunit over time. Catalytic immunoproteasome subunits were knocked out using different single guide RNAs (5 specific for PSMB8; 3 specific for PSMB9 and PSMB10, respectively) in human KMT2A-r AML cells (MOLM-13) and the chimerism of knockout and wildtype cells over time is visualized in the graphs. Each blue line represents the chimerism at day 0, 3, 6, 9, and 12. A decrease in the % of RFP+ cells (shown on the Y axis) over time, as seen most pronounced for PSMB8, reflects a competitive disadvantage of cells that harbor the respective knockout. Single guide RNAs (red lines) against the essential gene RPA3 were used as a positive control and non-targeting guide against Luciferase as a negative control (black line). (D) Percentage of Annexin+ cells (containing Annexin+-SYTOX®Blue+ and Annexin+-SYTOX®Blue- populations measured by flow cytometry) in KMT2Ar cell lines (MOLM-13, THP-1, MONO-MAC-6, KOPN-8, ML-2) transduced with shRNAs targeting PSMB8 or a non-targeting control (shNT) at day 6 post-infection. n=3-5 independent experiments; mean with SD; paired Student t test. (E) Growth curves depicting cell counting after trypan blue exclusion of MOLM-13 cells and MOLM-13 PSMB8 overexpressing cells (+PSMB8) transduced with shRNAs targeting PSMB8 or a non-targeting control (shNT). n=3 independent experiments; mean with SD. (F) Repres [file 12943_2023_1907_MOESM1_ESM.docx]

**Supplementary Material and Methods**

**Genome-wide CRISPR/Cas9 screening.** The human lentiviral CRISPR/Cas9 library was developed and kindly provided by Dr. X. S. Liu (Addgene, #1000000132). For the PR-957 screen the H2 library, containing 92817 guide RNAs targeting against 18436 genes was used. Guide-RNAs were designed using an improved algorithm and cloned into the lentiCRISPRv2 vector backbone (Addgene: #52961). The plasmid-library was electroporated into competent *E. coli Stbl4* and sequenced after Maxiprep to ensure a near-complete representation of guide-RNAs and homogeneous distribution of single guides (Gini-index at 0.1). For the *in vitro* screen, lentivirus was produced using HEK-293T cells transfected with the pooled library as well as the respective packaging plasmids (3ug pooled library, 3ug pMD2.G (Addgene, #12259), 3ug psPAX2 (Addgene, #12260) using FuGENE according to the manufacturer’s instructions. Lentivirus was harvested 48h and 58h after transfection, filtered (0.45um), pooled, snap-frozen and stored at -80C. To ensure a low MOI (1 single guide per gene in >90% of cells), the lentivirus was titrated on MOLM-13 cells to a transduction efficiency of 20-25%. To transduce the genome-scale CRISPR/Cas9 library at a proper representation into MOLM-13 cells, 6x 10^8^ cells were spinfected (872 g, 37C, 2h) in 6-well plates containing 4ml virus-containing cell culture medium and 1x 10^7^ cells per well. After the spin cells were transferred to T175 cell culture flasks and the virus-containing medium was diluted 1:1 with fresh complete medium (RPMI+10% FBS). After overnight incubation cells were washed from the viral particles and cultured in complete medium in T175 flasks at a density of 1x 10^6^/ml for 2 days. Subsequently, puromycin was added at a concentration of 1.5ug/ml and cells were selected for 4 days. After selection of guide-expressing cells the percentage of dead cells as well as the total number of living cells were determined by flow cytometry. To maintain representation, 4x 10^7^ cells per replicate have been assigned to the PR-957 treatment or diluent control (DMSO) groups of the screen (4 replicates per group). 5x 10^7^ cells were purified from apoptotic cells using the AnnexinV-based EasySep™ Dead Cell Removal Kit (Stem Cell Technologies) and snap-frozen as a pellet (baseline-sample). Cells in the PR-957-arm of the screen were treated with increasing concentrations of the drug (50-200nM) until a clear decline in total viable cell numbers was observed and the IC50 was reached. During the screen, cells were splitted to contain a minimum number of 4x 10^7^ viable cells in every single replicate. The cell density was constantly maintained between 0.5 and 1.5x 10^6^ cells per ml. On day 12 after start of PR-957 treatment cells were harvested and apoptotic cells were removed. Cell pellets of 5x 10^7^ viable cells were snap-frozen and stored at -80C. Genomic DNA was isolated from these pellets using phenol-chloroform extraction and subsequently eluted in TE buffer. Concentration was determined by Nanodrop. Library preparation was performed according to standard protocols (https://www. addgene.org/pooled-library/liu-crispr-knockout/).

**Global proteome analysis.** For library creation, the DDA and DIA data were searched independently using Pulsar in Spectronaut Professional+ (version 11.0.15038, Biognosys AG, Schlieren, Switzerland). The data were searched against a species specific (*Mus musculus* or *Homo sapiens*) Swissprot database. The data were searched with the following modifications: Carbamidomethyl (C) (Fixed) and Oxidation (M)/ Acetyl (Protein N-term) (Variable). A maximum of 2 missed cleavages for trypsin were allowed. The identifications were filtered to satisfy FDR of 1 % on peptide and protein level. For each species analysis, a DpD (DDA plus DIA) library was then created by merging the respective DDA and DIA libraries together in Spectronaut. These libraries contained 47291 (mouse); 45357 precursors, corresponding to 3781 (mouse); 3580 protein groups using Spectronaut protein inference. Relative quantification was performed in Spectronaut for each pairwise comparison using the replicate samples from each condition. The data (candidate table) and data reports (protein quantities) were then exported, and further data analyses and visualization were performed with R-studio (version 0.99.902) (http://www.R-project.org/.) using in-house pipelines and scripts.

**RNA-sequencing analysis.** RNA was extracted using the RNeasy MinElute Cleanup Kit (Qiagen) following the manufacturer´s instructions. RNA-seq libraries were generated using the TruSeq RNA Library Prep Kit (Illumina, San Diego, CA, USA). Sequencing was performed at Genewiz (NovaSeq 6000, 150bp, paired-end). For analysis, reads were aligned to the human reference genome hg38 using STAR. Quantification of the reads was performed by featureCounts, followed by normalization, transformation and differential gene expression analysis in DEseq2. GSEA was carried out on a public server "Gene Pattern" of Broad Institute.

**ATAC-, Cut&Tag-, Cut&Run-sequencing analysis.** Sequencing was performed at Genewiz (NovaSeq 6000, 150bp, paired-end; Illumina) or on a NextSeq 550 System (Illumina). For analysis, reads were aligned to the human reference genome hg38 using Bowtie2. Peak calling was done using MACS2 or SEACR. Normalization, transformation and differentially accessible regions analysis for ATAC-seq was done with DEseq2. Quantification of the TSS regions for Cut&Run was performed with Bedtools.

**CRISPR/Cas9 Screening analysis.** For analysis of the genetic CRISPR/Cas9 screen, next generation sequencing was performed on an Illumina NextSeq platform (75bp, single reads) aiming for a minimum of 30Mio reads per sample. Alignment and statistical analysis of the data was conducted using the MAGeCK and MAGeCK-Flute algorithms. Specifically, MAGeCK was used to align guide sequences from FASTQ files based on the guide-matrix. Subsequently, the MAGeCK-MLE algorithm was used to statistically compare dropout and enrichment of guides between day 0 and day 12 separately for treated (PR-957) versus untreated (DMSO) conditions. Finally, the FluteMLE algorithm was utilized to identify functional genetic vulnerabilities upon PR-957 treatment based on the MAGeCK-MLE output.

**Spleen colony formation in vivo assays (CFU-S12):** Bone marrow cells were collected from donor mice and 1x10^2^ LSK cells were FACS sorted and injected via tail vein into lethally irradiated (12 Gy TBI) C57BL/6 recipient mice. At day 12 post-injection, spleens from recipient mice were harvested and stained with Bouin’s fixative solution (Sigma-Aldrich), and colonies were counted as previously described (Purton LE & Scadden DT. Cell Stem Cell. 2007).

**Rescue experiment**

For the PSMB8 rescue experiment, base-optimized human PSMB8 cDNA was cloned into the pLEX vector and transduced into targeted cells. Genetic inactivation by RNAi was then performed as explained in the main Material and Methods part.

**Supplementary Figures**

**
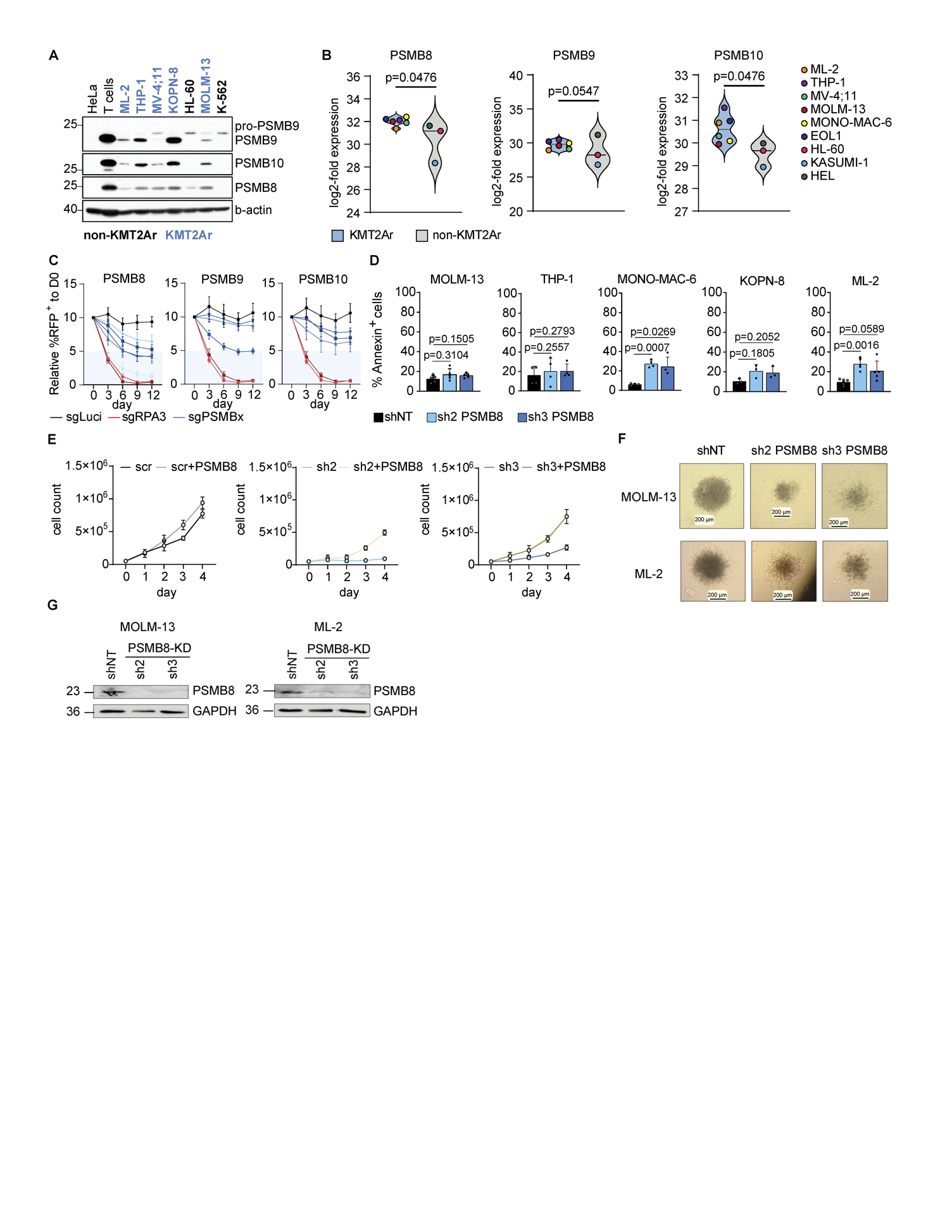
**

**Fig. S1.** (**A**) Western Blotting showing expression of PSMB8, PSMB9 and PSMB10 in KMT2A-r (ML-2, THP-1, MV-4;11, KOPN-8, MOLM-13) and non-KMT2A-r (HL-60, K-562) cell lines. Hela cells were used as a negative control, since they do not express immunoproteasome, and T cells as a positive control. (**B**) Violin plots displaying log2-fold protein expression as assessed by proteome analysis (Jayavelu et al., Cancer Cell, 2022; PXD022894) of PSMB8, PSMB9 and PSMB10 in different KMT2A-r and non-KMT2A-r cell lines. Mann-Whitney U test. (**C**) CRISPR-Cas9 cell competition assay showing the effect of deletion of each catalytic immunoproteasome subunit over time. Catalytic immunoproteasome subunits were knocked out using different single guide RNAs (5 specific for PSMB8; 3 specific for PSMB9 and PSMB10, respectively) in human KMT2A-r AML cells (MOLM-13) and the chimerism of knockout and wildtype cells over time is visualized in the graphs. Each blue line represents the chimerism at day 0, 3, 6, 9, and 12. A decrease in the % of RFP+ cells (shown on the Y axis) over time, as seen most pronounced for PSMB8, reflects a competitive disadvantage of cells that harbor the respective knockout. Single guide RNAs (red lines) against the essential gene RPA3 were used as a positive control and non-targeting guide against Luciferase as a negative control (black line). (**D**) Percentage of Annexin^+^ cells (containing Annexin^+^-SYTOX^®^Blue^+^ and Annexin^+^-SYTOX^®^Blue^-^ populations measured by flow cytometry) in KMT2Ar cell lines (MOLM-13, THP-1, MONO-MAC-6, KOPN-8, ML-2) transduced with shRNAs targeting PSMB8 or a non-targeting control (shNT) at day 6 post-infection. n=3-5 independent experiments; mean with SD; paired Student t test. (**E**) Growth curves depicting cell counting after trypan blue exclusion of MOLM-13 cells and MOLM-13 PSMB8 overexpressing cells (+PSMB8) transduced with shRNAs targeting PSMB8 or a non-targeting control (shNT). n=3 independent experiments; mean with SD. (**F**) Representative pictures of colonies from MOLM-13 and ML-2 cells transduced with PSMB8 shRNAs or shNT. Scale bars, 200 µm. Representative Western Blotting plots of MOLM-13 and ML-2 cells confirming PSMB8 deletion at day 4 post-infection before transplantation into NSGS recipient mice. **(G)** Representative Western Blotting plots of MOLM-13 and ML-2 cells confirming PSMB8 deletion at day 4 post-infection before transplantation into NSGS recipient mice.


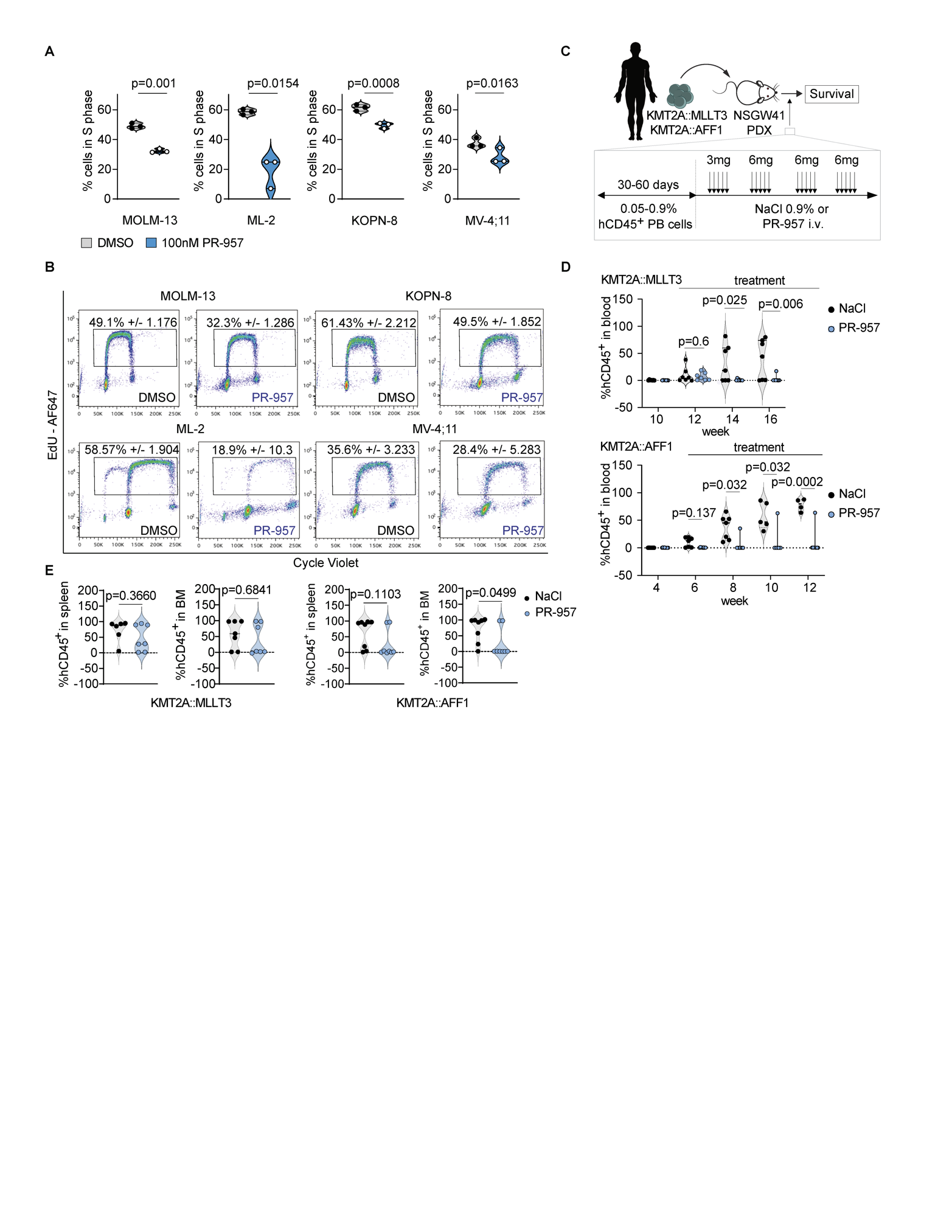


**Fig. S2.** (**A**) Violin plots showing the percentage of cells detected in S phase in MOLM-13, ML-2, KOPN-8 and MV-4;11, cells after treatment with 100nM PR-957 or DMSO for 24 hours. Samples were labeled for flow cytometry-based analysis of cell cycle using the Click-iT® EdU assay. n=3 independent experiments; paired Student t test. (**B**) Representative flow cytometry plots from Click-iT® EdU assay. (**C**) Schematic representation of patient derived xenografts (PDX). (**D**) Violin plots depicting % of hCD45+ cells in peripheral blood in PDX models of KMT2A::MLLT3 and KMT2A::AFF1. 2-way ANOVA. (**E**) Violin plots of hCD45+ cells in spleen and bone marrow at the time of sacrifice. Mann-Whitney U test.

**
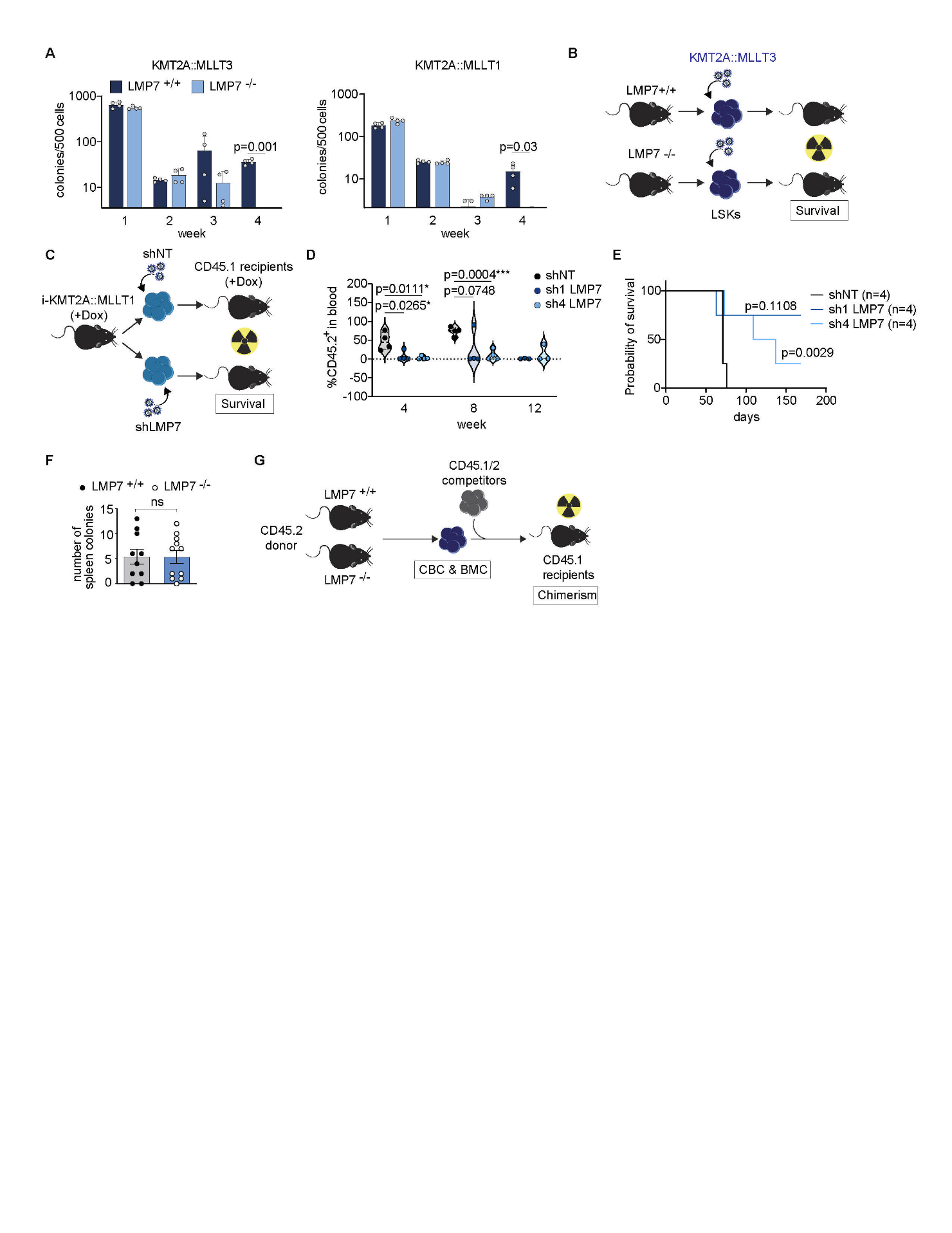
**

**Fig. S3.** (**A**) Serial re-plating to assess colony formation in methylcellulose using murine LSK (Lin^-^ Sca1^+^ c-Kit^+^) cells isolated from LMP7^+/+^ or LMP7^-/-^ mice and transformed with KMT2A::MLLT3 or KMT2A::MLLT1. n=4 independent experiments; mean with SD; paired Student t test. (**B**) Schematic representation showing the transplantation of KMT2A::MLLT3 retrovirally transformed LSKs from LMP7^+/+^ or LMP7^-/-^ mice. (**C**) Schematic representation describing the transplantation of i-KMT2A::MLLT1 BM cells lentivirally transduced with shRNA1 or shRNA4 against LMP7 or a non-targeting control (shNT) into CD45.1 recipient mice. Diet of the donor mice was supplemented with Doxycycline (DOX; 0,545 g/kg) for 2 weeks to induce KMT2A::MLLT1 expression. Recipient mice were also kept with DOX supplemented food. (**D**) Violin plots depicting percentage of CD45.2+ cells in peripheral blood of CD45.1 recipient mice from sh1 LMP7 (n=4), sh4 LMP7 (n=4) or shNT (n=4) transformed i-KMT2A::MLLT1. One cohort; 2-way ANOVA. (**E**) Kaplan-Meier survival curves of CD45.1 recipient mice transplanted with 1x10^6^ cells as shown in S3C. One cohort; Mantel-Cox test. (**F**) Spleen Colony Formation Assay in vivo (CFU-S12): Spleen colony numbers 12 days after injection of 100 LSK cells isolated from LMP7^+/+^ (n=10) or LMP7^-/-^ (n=10) mice. Two independent cohorts; Mann-Whitney U test (**G**) Schematic representation depicting competitive repopulation assay to investigate the effects of LMP7 depletion on normal hematopoietic stem- and progenitor cells (HSPCs).

**
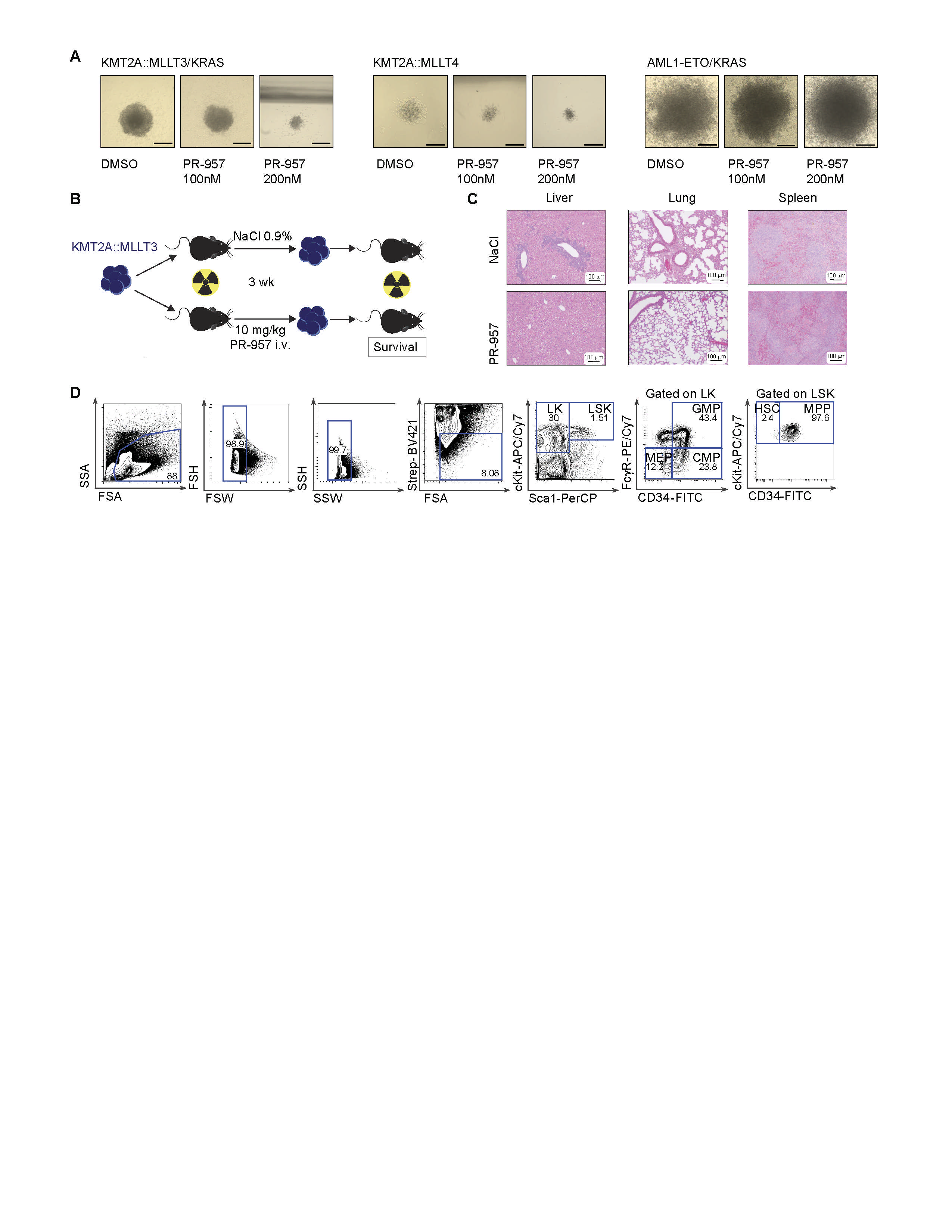
**

**Fig. S4.** (**A**) Representative pictures of colonies from murine LSK cells transformed with KMT2A::MLLT3/KRAS, KMT2A::MLLT4 or AML1-ETO/KRAS at week 2. Scale bars, 200 µm. (**B**) Schematic representation of *in vivo* PR-957 vs NaCl 0.9% treatment in C57BL/6 mice transplanted with KMT2A::MLLT3 leukemic cells. Subsequent secondary transplantation of whole bone marrow cells into secondary recipients. (**C**) Pictures of tissue sections from liver, lung and spleen of *in vivo* PR-957 vs NaCl treated mice at the time of sacrifice. (**D**) Representative flow cytometry plots with the gating strategy for the analysis of granulocyte-macrophage progenitors (GMPs), megakaryocyte-erythroid progenitors (MEPs), common myeloid progenitors (CMPs), hematopoietic stem cells (HSCs) and multipotent progenitors (MPPs) in the competitive repopulation assay. FSA: Forward Scatter Area; SSA: Side Scatter Area; FSH: Forward Scatter Height; FSW: Forward Scatter Width; SSH: Side Scatter Height; SSW: Side Scatter Width; LK: Lineage- cKit+; LSK: Lineage- Sca1+ cKit+.


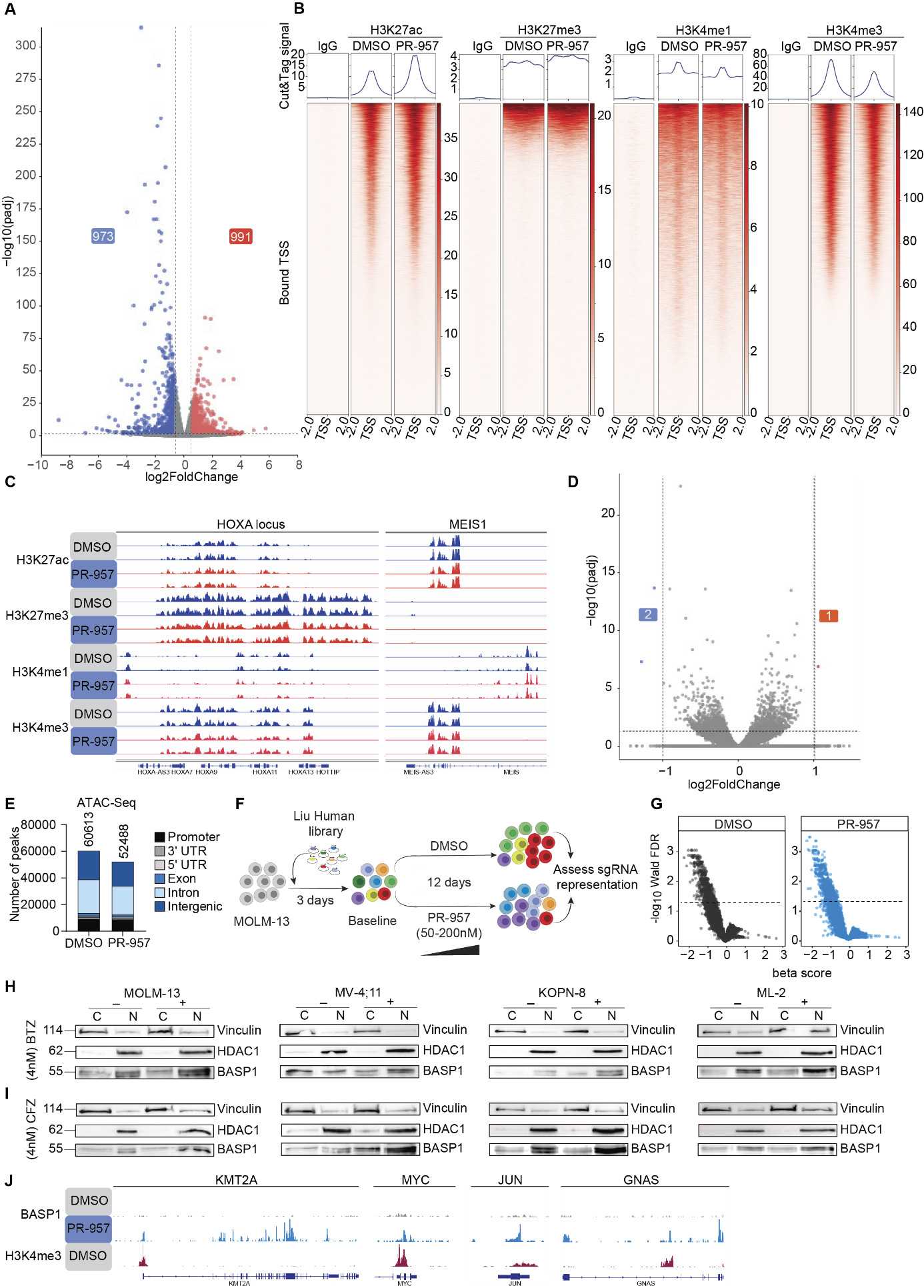


**Fig. S5.** (**A**) Volcano plot of differentially regulated genes in RNA-sequencing. 100nM PR-957 vs. DMSO, 72h, MOLM-13. Upregulated (red; FC>1.5, p<0.05) and downregulated (blue; FC<-1.5, p<0.05). (**B**) Heatmaps displaying H3K27ac, H3K27me3, H3K4me1 and H3K4me3 Cut&Tag signal mapping to a 2-kb window around TSS. 100nM PR-957 vs. DMSO, 48h, MOLM-13. (**C**) Integrative Genomics Viewer (IGV) tracks from Cut&Tag-sequencing data in MOLM-13 cells depicting binding of H3K27ac, H3K27me3, H3K4me1 and H3K4me3 after DMSO or PR-957 treatment at HOXA and MEIS1 loci. (**D**) Volcano plot of differentially accessible regions in ATAC-seq. 100nM PR-957 vs. DMSO, 72h, MOLM-13. Upregulated (red; FC>2, p<0.05) and downregulated (blue; FC<-2, p<0.05). (**E**) Stacked bar plot depicting genomic distribution of PSMB8 ATAC-seq peaks. 100nM PR-957 vs. DMSO, 72h, MOLM-13. (**F**) Schematic representation of the genome-wide CRISPR-Cas9 screen. (**G**) Volcano plots showing the distribution of genes being enriched (positive beta-scores) or depleted (negative beta-scores) in the genome-wide CRISPR-Cas9 screen in PR-957 treated MOLM-13 cells and DMSO diluent control. (**H-I**) Western Blotting showing expression of BASP1 in nuclear (N) and cytoplasmic (C) fractions of MOLM-13, MV-4;11, KOPN-8 and ML-2 cells. (**H**) BTZ (4nM) vs. DMSO, 72h. (**I**) CFZ (4nM) vs. DMSO, 72h. (**J**) Integrative Genomics Viewer (IGV) tracks from Cut&Run-sequencing data in MOLM-13 cells depicting binding of BASP1 after DMSO or PR-957 treatment and of H3K4me3 after DMSO treatment at the KMT2A, MYC, JUN and GNAS loci.


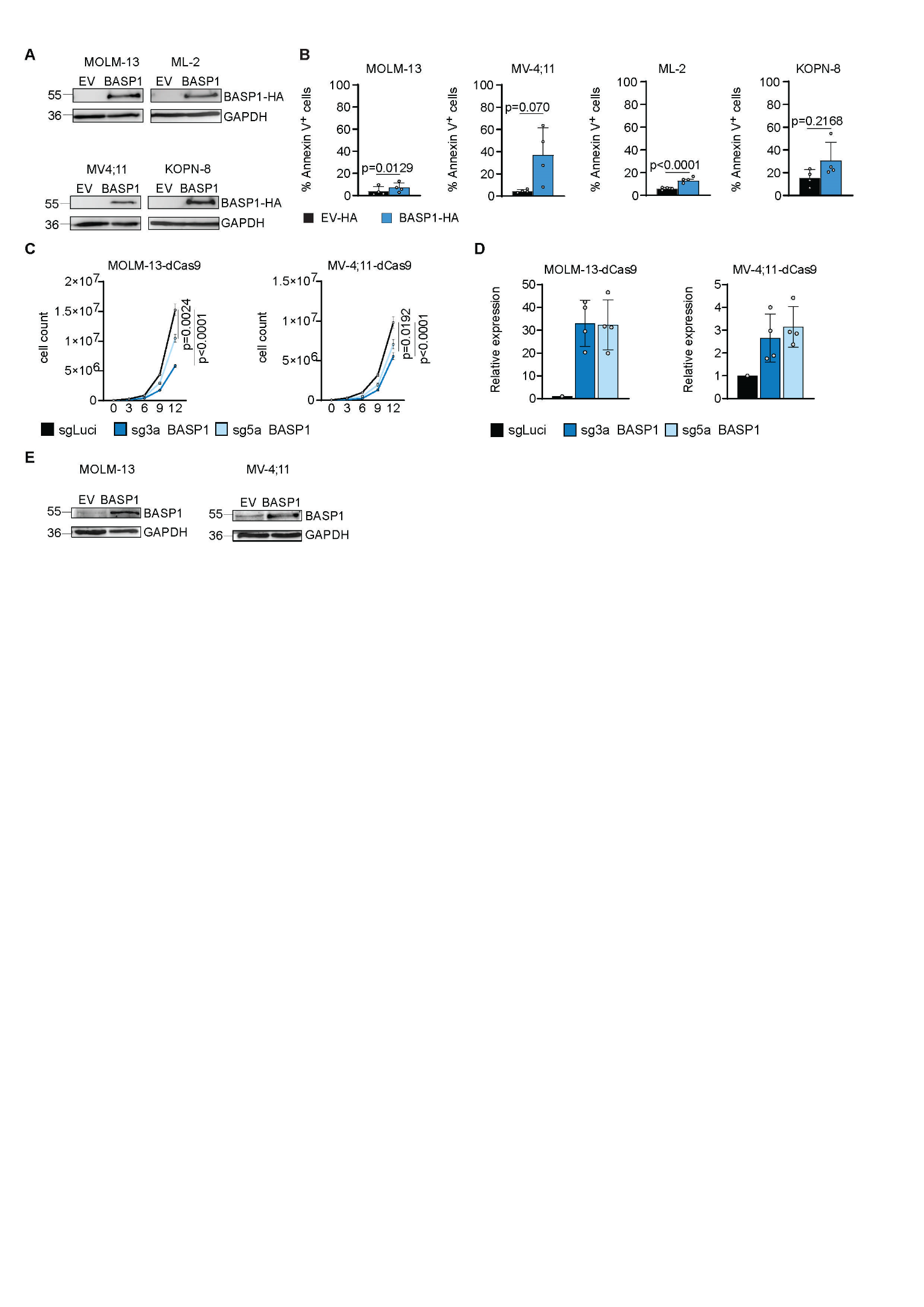


**Fig. S6.** (**A**) Western Blotting confirming BASP1 overexpression after transduction with a pLEX vector containing the sequence of human BASP1 with an HA tag (BASP1) or an empty vector with the HA tag (EV) as a control in MOLM-13, MV-4;11, ML-2 and KOPN-8 cells. Samples were collected at day 6 post-infection. (**B**) Percentage of Annexin^+^ cells of KMT2A-r cell lines overexpressing BASP1 or control cells at day 6 post-infection. n=4 independent experiments; mean with SD; paired Student t test. (**C**) Growth curves of MOLM-13-deadCas9 (MOLM-13-dCas9) and MV-4;11-dCas9 cells containing the MS2-P65-HSF1 activator helper complex transduced with sgRNAs designed to target the promoter region of BASP1 (sgRNA3a BASP1 and sgRNA5a BASP1) or sgRNA Luciferase as a negative control. n=4 independent experiments; mean with SD; 2-way ANOVA. (**D**) Relative BASP1 mRNA expression in BASP1-overexpressing cells (MOLM-13-dCas9 and MV-4;11-dCas9) compared to sgRNA Luciferase cells assessed by Real Time Quantitative PCR (RT-qPCR). n=4 independent experiments; mean with SD. (**E**) Western Blotting confirming BASP1 overexpression after transduction with pLEX-BASP1 (BASP1) or pLEX-EV (EV) in MOLM-13 and MV-4;11 cells. Samples were collected 96 hours after transduction and before transplantation into recipient mice.


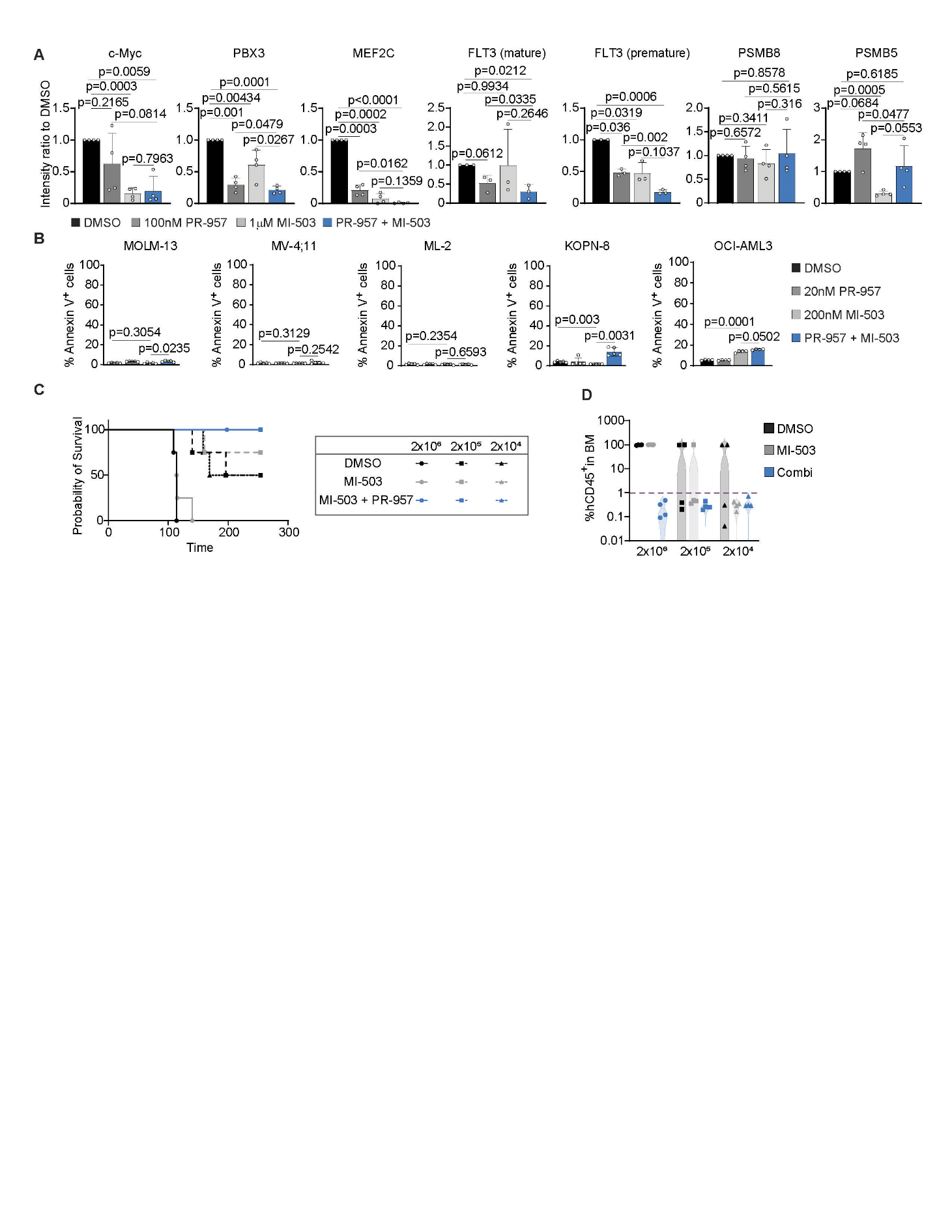


**Fig. S7.** (**A**) Western Blotting quantification of c-MYC, MEF2C, FLT3 (mature), FLT3 (premature), PBX3, PSMB5 and PSMB8 protein expression in MOLM-13 cells treated with 100nM PR-957, 1μM MI-503, a combination of both or DMSO. n=3-4 independent experiments; mean with SD; paired Student t test, (**B**) Percentage of Annexin^+^ cells in MOLM-13, MV-4;11, ML-2, KOPN-8 and OCI-AML3 cells after treatment with 20nM PR-957, 200nM MI-503, a combination of both or DMSO as diluent control at day 8 post-infection. n=5 independent experiments; mean with SD; paired Student t test. (**C**) Kaplan-Meier survival curves of NXG recipient mice transplanted with limiting numbers (2x10^6^, 2x10^5^, 2x10^4^) of whole bone marrow cells from *in vivo* treated mice (DMSO, MI-503, MI-503 + PR-957). Two independent cohorts; Mantel-Cox test. (**D**) Violin plots showing percentage of hCD45^+^ cells in bone marrow (BM) at the time of sacrifice.

**
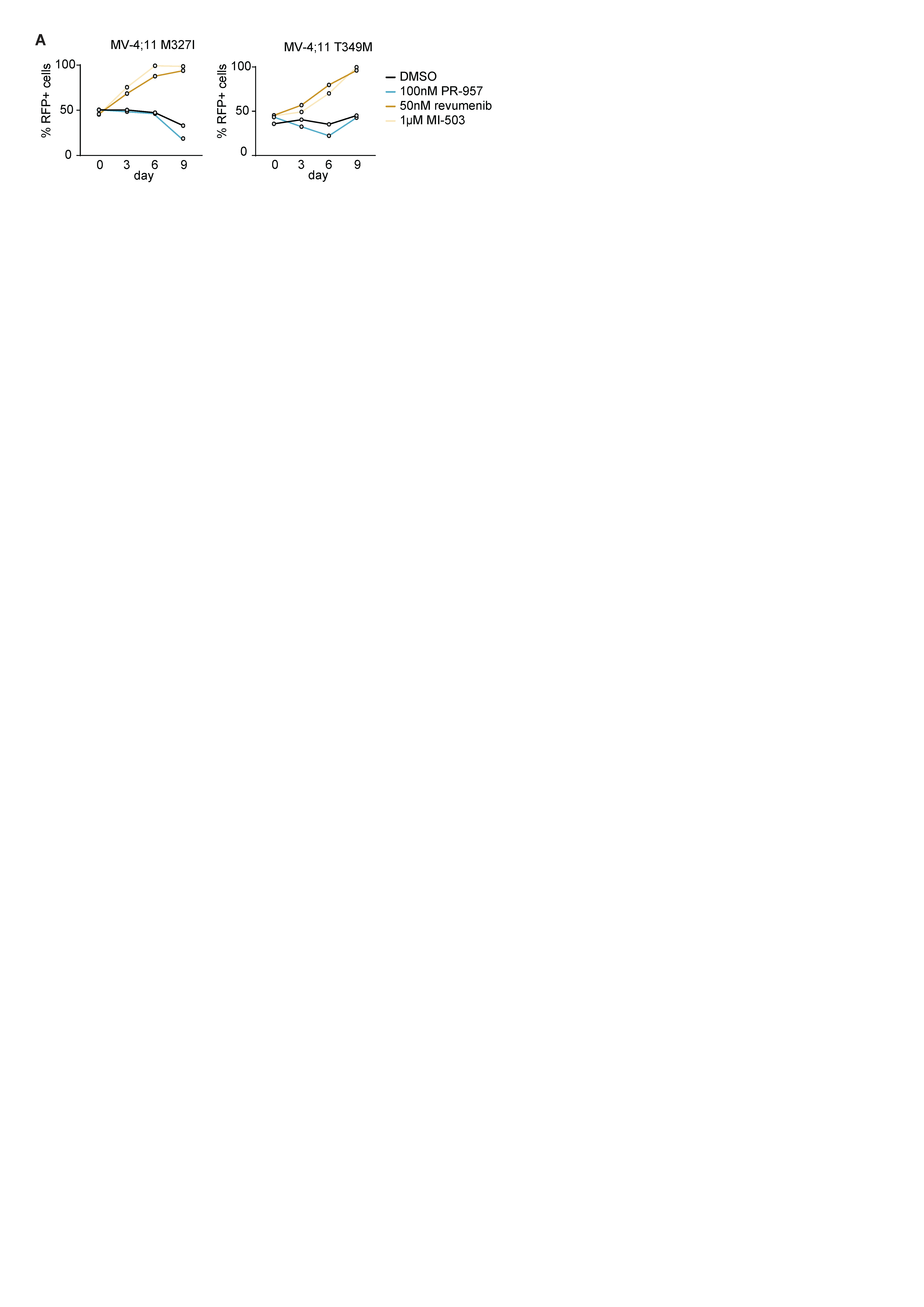
**

**Fig. S8.** (**A**) Negative selection cell competition assay using MV-4;11 Menin-wildtype (BFP^+^) cells and resistant MV4;11 Menin M327I or T349M (RFP^+^) cells (as published in Perner et al. Nature 2023). Chimerism of RFP^+^ mutant clones over BFP^+^ Menin-wildtype cells is visualized for a total of 9 days upon exposure to PR-957(100nM), Revumenib (50nM), MI503 (1μM) or DMSO as a control.

**Supplementary Tables**

**Table S1.** Flow cytometry and western blot antibodies used in this study.

| **Antibody** | **Company** | **Cataloge number** |
| --- | --- | --- |
| APC-Cy7 anti-mouse CD11b | BioLegend | Cat #: 101225 |
| APC anti-mouse Gr-1 | BioLegend | Cat #: 108412 |
| Pacific Blue anti-mouse Gr-1 | BioLegend | Cat #: 108430 |
| APC-Cy7 anti-mouse CD3 | BioLegend | Cat #: 100221 |
| PE anti-mouse CD3 | BioLegend | Cat #: 100205 |
| PE-Cy7 anti-mouse CD19 | BioLegend | Cat #: 115519 |
| APC-Cy7 anti-mouse cKit | BioLegend | Cat #: 105826 |
| APC anti-mouse cKit | BioLegend | Cat #: 161505 |
| PE anti-mouse Sca1 | BioLegend | Cat #: 108107 |
| PerCP-Cy5.5 anti-mouse Sca1 | BioLegend | Cat #: 108123 |
| FITC anti-mouse CD34 | BD Biosciences | Cat #: 553733 |
| PE-Cy7 anti-mouse FcRγ | BioLegend | Cat #: 101318 |
| PerCP-Cy5.5 anti-mouse CD150 | BioLegend | Cat #: 115921 |
| APC-Cy7 anti-mouse CD48 | BioLegend | Cat #: 103431 |
| APC anti-mouse CD45.2 | BioLegend | Cat #: 109814 |
| PE anti-mouse CD45.1 | BioLegend | Cat #: 110708 |
| BV421 anti-Streptavidine | BioLegend | Cat #: 405226 |
| Biotin anti-mouse CD3ε | BioLegend | Cat #: 100304 |
| Biotin anti-mouse CD19 | BioLegend | Cat #: 115503 |
| Biotin anti-mouse Gr-1 | BioLegend | Cat #: 108404 |
| Biotin anti-mouse TER119 | BioLegend | Cat #: 116204 |
| Biotin anti-mouse B220 | BioLegend | Cat #: 103203 |
| Biotin anti-mouse CD4 | BioLegend | Cat #: 100404 |
| Biotin anti-mouse CD8 | BioLegend | Cat #: 100704 |
| Biotin anti-mouse IL7Ra | BioLegend | Cat #: 121104 |
| anti-BASP1 | Thermo Fisher Scientific | Cat #: 703692 |
| anti-c-Myc | Abcam | Cat #: ab32072 |
| anti-FLT3 | Cell Signaling | Cat #: CS3462 |
| anti-GAPDH | Meridian Life Sciences | Cat #: H68504M |
| anti-HA-Tag | Cell Signaling | Cat #: CS3724 |
| anti-HDAC1 | Cell Signaling | Cat #: CS5356 |
| anti-MEF2C | Cell Signaling | Cat #: CS5030S |
| anti-PBX3 | Abcam | Cat #: ab109173 |
| anti-PSMB5 | Santa Cruz Biotechnology | Cat #: sc393931 |
| anti-PSMB8/LMP7 | Abcam | Cat #: ab3329 |
| anti-PSMB8/LMP7 | Santa Cruz Biotechnology | Cat #: sc365699 |
| anti-PSMB9/LMP2 | (30) |  |
| anti-PSMB10/MECL1 | Thermo Fisher Scientific | Cat #: PA5-19146 |
| anti-Vinculin | Sigma Aldrich | Cat #: V9131 |
| anti-β-actin | Santa Cruz Biotechnology | Cat #: sc47778 |

**Table S2.** shRNA sequences used in this study.

| **shRNA name** | **Sequence (5´-3´)** |
| --- | --- |
| shNT | CCGGGCGCGATAGCGCTAATAATTTCTCGAGAAATTATTAGCGCTATCGCGCTTTTT |
| human PSMB8 shRNA2 | CCGGCCTCTCTATGGGCAGTATGATCTCGAGATCATACTGCCCATAGAGAGGTTTTT |
| human PSMB8 shRNA3 | CCGGCCACTCACAGAGACAGCTATTCTCGAGAATAGCTGTCTCTGTGAGTGGTTTTT |
| mouse LMP7 shRNA1 | CCGGGGCCGCAGAGCTATTGCTTATCTCGAGATAAGCAATAGCTCTGCGGCCTTTTT |
| mouse LMP7 shRNA4 | CCGGGAAAGTGGAGAGTTCCGATGTCTCGAGACATCGGAACTCTCCACTTTCTTTTT |

**Table S3.** sgRNAs for CRISPRa used in this study.

| **sgRNA name** | **Sequence (5´-3´)** |
| --- | --- |
| Luciferase sgRNA | GATTCTAAAACGGATTACCA |
| sgRNA3a BASP1 | CGGGGAGCGCGGGAGGAGGG |
| sgRNA5a BASP1 | GGGCGGGGAGCGCGGGAGGA |

**Table S4.** Primer sequences for RT-qPCR used in this study.

| **Primer name** | **Sequence (5´-3´)** |
| --- | --- |
| BASP1_Fw | AGGGGAACCAAAAAGACTGA |
| BASP1_Rv | GGTGTGGAACTAGGCGCTTC |
| PSMB8_Fw | CCTTACCTGCTTGGCACCATG |
| PSMB8_Rv | TTGGAGGCTG-CCGACACTGAA |
| B2M_Fw | tgtgtctgggtttcatccatccga |
| B2M_Rv | cacacggcaggcatactcatcttt |
